# Macrophage activation by IFN-γ triggers restriction of phagosomal copper from intracellular pathogens

**DOI:** 10.1371/journal.ppat.1007444

**Published:** 2018-11-19

**Authors:** Qian Shen, Matthew J. Beucler, Stephanie C. Ray, Chad A. Rappleye

**Affiliations:** Department of Microbiology, Ohio State University, Columbus, OH, United States of America; University of Georgia, UNITED STATES

## Abstract

Copper toxicity and copper limitation can both be effective host defense mechanisms against pathogens. Tolerance of high copper by fungi makes toxicity as a defense mechanism largely ineffective against fungal pathogens. A forward genetic screen for *Histoplasma capsulatum* mutant yeasts unable to replicate within macrophages showed the Ctr3 copper transporter is required for intramacrophage proliferation. Ctr3 mediates copper uptake and is required for growth in low copper. Transcription of the *CTR3* gene is induced by differentiation of *H*. *capsulatum* into pathogenic yeasts and by low available copper, but not decreased iron. Low expression of a *CTR3* transcriptional reporter by intracellular yeasts implies that phagosomes of non-activated macrophages have moderate copper levels. This is further supported by the replication of Ctr3-deficient yeasts within the phagosome of non-activated macrophages. However, IFN-γ activation of phagocytes causes restriction of phagosomal copper as shown by upregulation of the *CTR3* transcriptional reporter and by the failure of Ctr3-deficient yeasts, but not Ctr3 expressing yeasts, to proliferate within these macrophages. Accordingly, in a respiratory model of histoplasmosis, Ctr3-deficient yeasts are fully virulent during phases of the innate immune response but are attenuated after the onset of adaptive immunity. Thus, while technical limitations prevent direct measurement of phagosomal copper concentrations and copper-independent factors can influence gene expression, both the *CTR3* promoter induction and the attenuation of Ctr3-deficient yeasts indicate activation of macrophages switches the phagosome from a copper-replete to a copper-depleted environment, forcing *H*. *capsulatum* reliance on Ctr3 for copper acquisition.

## Author summary

Control of primary pathogens that infect phagocytes often requires adaptive immunity, but the mechanisms that convert host cells from permissive to antimicrobial states are only partially understood. The intracellular fungal pathogen *Histoplasma capsulatum* resides and proliferates within the macrophage phagosome. During innate immunity, macrophages which normally control fungi prove ineffective against *H*. *capsulatum* yeasts. At this stage, the phagosome of unactivated macrophages has ample copper that facilitates intracellular growth of *Histoplasma* but does not cause copper toxicity. However, the onset of adaptive immunity and the subsequent activation of macrophages decreases phagosomal copper and macrophages become less permissive to *Histoplasma* proliferation. IFN-γ acts as a key cytokine for switching the macrophage strategy by changing phagosomes from a copper-sufficient to a copper-depleted state in order to control intracellular pathogens. In such activated macrophages, *H*. *capsulatum* yeasts upregulate expression of the Ctr3 copper transporter to enable continued acquisition of essential copper.

## Introduction

To successfully infect and colonize a host, pathogens must acquire sufficient nutrients from the host to enable microbe growth and proliferation. These metabolic resources include, but are not limited to, essential metals. The nutrient-limited phagosome represents a particularly challenging environment for intracellular pathogens as mammalian hosts can sequester essential elements such as iron and zinc from pathogens. This has been termed “nutritional immunity” [1,2]. For example, host molecules such as heme, ferritin, transferrin, and lactoferrin make iron largely inaccessible to microbes [1]. However, successful pathogens have developed sophisticated strategies to combat iron limitation. For example, *Mycobacterium tuberculosis* and the fungal pathogen *Histoplasma capsulatum* secrete iron-chelating siderophores [3–5]. Accordingly, inability to synthesize siderophores severely impairs intracellular growth [5,6]. In addition, *H*. *capsulatum* maintains a slightly acidic intra-phagosomal pH which is sufficient to release iron from host transferrin [7]. Mammalian hosts also restrict available zinc by production of zinc chelating proteins such as S100 family proteins and calprotectin [8,9]. In addition, host zinc transporters (ZIPs) are employed to tightly control zinc levels in different cellular compartments [10]. Host zinc limitation mechanisms are an important aspect of activation of cellular immunity [11]. However, as with iron limitation, some pathogens have evolved efficient mechanisms to counteract zinc sequestration. High affinity transporters expressed by *Salmonella* species and *H*. *capsulatum* (ZnuABC and Zrt2, respectively) enable these pathogens to import zinc in environments with low zinc concentrations [12–15]. Without these zinc transporters *Salmonella* and *H*. *capsulatum* intracellular proliferation is significantly attenuated. Employing an alternative strategy, the fungal pathogen *Candida albicans* expresses zincophore (Pra1), a zinc-chelating molecule, to scavenge zinc during endothelial invasion [16].

Like iron and zinc, pathogen acquisition of copper during infection is essential, but high levels of copper are toxic. Copper killing mechanisms involve reactive oxygen-generating fenton-type reactions, nitrosative stress, or iron-sulfur cluster attack [1]. Recent evidence has shown that immune cells can utilize excessive copper as a powerful weapon to kill pathogens during innate immunity [17–19]. For pathogens, an inability to decrease cellular copper can impair pathogen virulence. For example, *M*. *tuberculosis* survival in host cells depends on copper exporter proteins [19] and *Salmonella* systemic infection requires detoxification of excess copper by a multi-copper-ion oxidase (CueO) [20]. The fungus *Cryptococcus neoformans (grubii)* utilizes copper-sequestering metallothionein (Cmt) proteins for full virulence during pulmonary infection [17]. On the other hand, there is evidence that host defenses also use copper limitation in some tissue environments. During kidney infection, *C*. *albicans* switches from copper-dependent superoxide dismutase 1 (Sod1) to expression of the copper-independent Sod3 [21]. Proliferation of *C*. *neoformans* in murine brains requires two copper transporters (Ctr1 and Ctr4) indicating that copper is limited in the mouse CNS [22,23]. Thus, maintenance of copper homeostasis in host environments with high or low copper environments is essential for pathogens to establish successful infections.

*H*. *capsulatum* is a primary fungal pathogen that is not efficiently controlled by innate immunity alone since clearance requires activation of cell-mediated immunity [24,25]. *H*. *capsulatum* resides within the phagosome of host phagocytes, an environment that is initially permissive for fungal proliferation. Through a forward genetic screen, we identified a homolog of copper transporters (Ctr3) which was required for growth of *H*. *capsulatum* in low copper and within the phagosome of host macrophages. We determined that Ctr3 enhances *H*. *capsulatum* survival in vivo specifically during the peak of the adaptive immune response to pulmonary infection. Consistent with this, expression of *CTR3* increases in low copper concentrations in vitro and in activated, but not in unactivated macrophages. These findings show that copper is sufficiently available to intramacrophage *H*. *capsulatum* during innate immunity, but that activation of macrophages induces copper limitation to enact fungal control.

## Results

### Intramacrophage growth of *H*. *capsulatum* requires Ctr3

To identify genes required for intramacrophage growth, a genetic screen was designed to identify mutants unable to proliferate within macrophages. Insertion mutants were created using *Agrobacterium tumefaciens*-mediated transformation of a T-DNA element previously shown to provide relatively random and trackable mutations [26]. To facilitate efficient identification of mutants with reduced intramacrophage proliferation, two indirect assays of *H*. *capsulatum* yeast replication within macrophages were used. First, increasing fluorescence of red-fluorescence protein (RFP) expressing yeasts was used to indicate intramacrophage yeast replication [27]. Second, a lacZ-expressing macrophage cell line was used to rapidly quantify the ability of mutant yeast to lyse infected macrophages as a result of yeast replication [28]. Individual *H*. *capsulatum* mutants were added to macrophage populations to initiate infections. RFP-fluorescence was monitored daily over 7–8 days after which remaining macrophages were quantified by the remaining β-galactosidase activity. Mutants were selected that showed less than 30% increase in RFP fluorescence and/or at least 30% reduction in macrophage lysis. Of 40,000 insertion mutants, 178 had reduced intramacrophage growth and/or attenuated virulence in macrophages.

**Fig 1:**
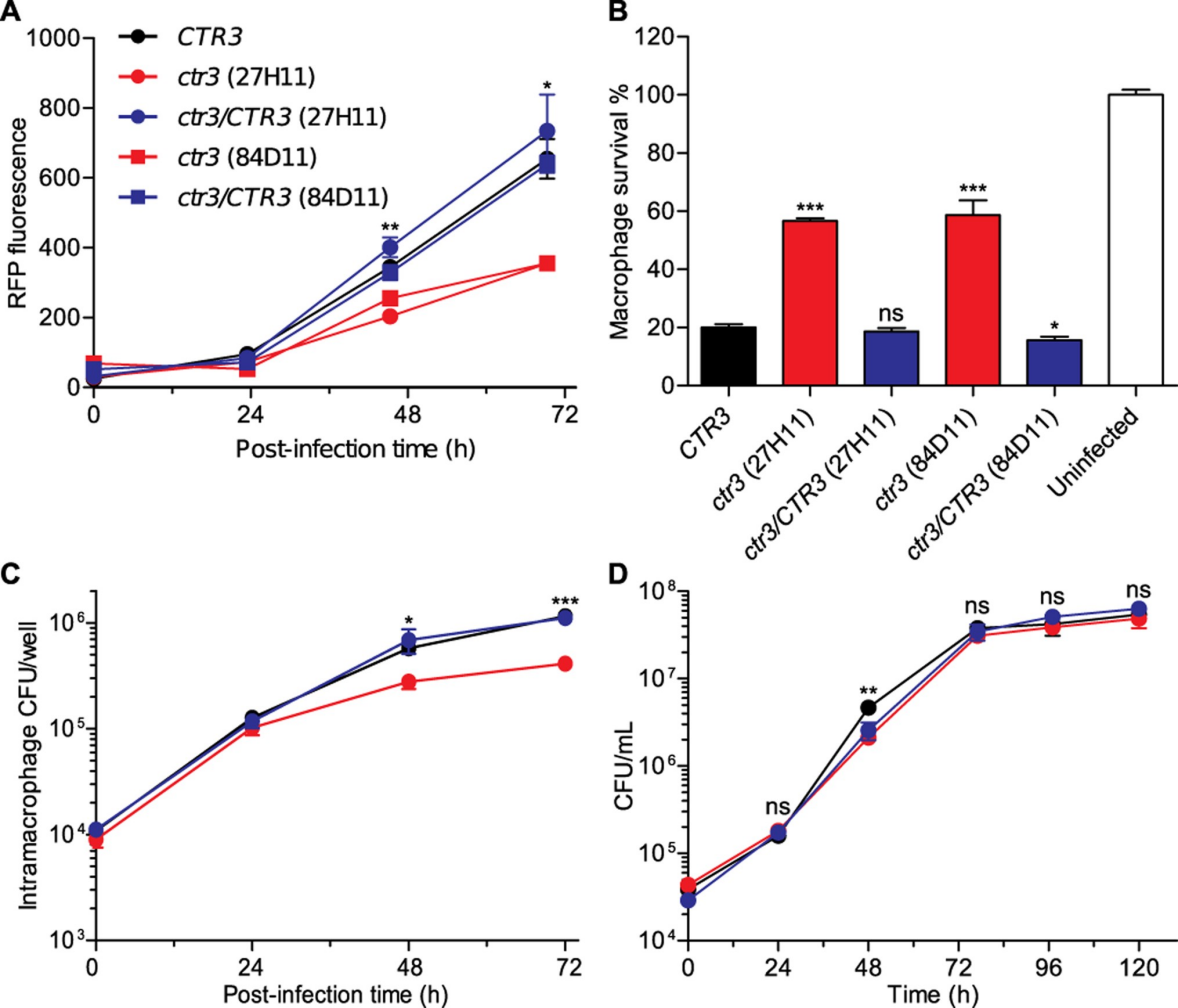
*H*. *capsulatum* intramacrophage growth requires Ctr3. *H*. *capsulatum* proliferation in macrophages was measured by fluorescence of RFP-expressing yeasts **(A)**, yeast-growth dependent lysis of the macrophage population **(B)**, and determination of intracellular viable yeasts **(C)**. **(A)** RFP-fluorescence of intracellular *H*. *capsulatum* yeasts was monitored over 72 hours following infection of P388D1 macrophages at an MOI of 1:2 with *CTR3*-expressing yeasts (*CTR3*; black), two mutants with the *CTR3* gene disrupted by T-DNA insertion (*ctr3* 27H11 (circles) and 84D11(squares); red), or mutant strains complemented with a wild-type *CTR3* gene (*ctr3/CTR3*; blue). **(B)** Survival of lacZ-expressing P388D1 macrophages was quantified after 7 days of infection by removal of culture medium and measurement of the remaining macrophage-derived β-galactosidase activity. **(C)** Viability of intracellular *H*. *capsulatum* yeasts over 72 hours was determined by lysing infected macrophages and plating of the lysate on solid HMM medium to enumerate colony forming units (CFU). **(D)** Growth of *CTR3*-expressing (black), *ctr3* mutant (27H11; red), and the *ctr3/CTR3* complemented (blue) strains in liquid culture in HMM at 37°C was measured by plating dilutions of the culture on solid medium to enumerate CFU. All data represent the average ± standard deviation of biological replicates (n = 3). Statistically significant differences between *CTR3* and *ctr3* strains were determined by one-tailed Student’s *t*-test and are indicated with asterisks (* *P* < 0.05, ** *P* < 0.01, *** *P* < 0.001).

The insertion mutations were mapped to the genome by sequencing the regions flanking the T-DNA insertion, and two mutants (27H11 and 84D11) were identified which had T-DNA insertions in the promoter region of a gene encoding a putative copper transporter (193 bp and 215 bp upstream of the CDS initiation codon for 27H11 and 84D11, respectively). We designated the gene *CTR3* based on phylogenetic analysis that showed the gene product was similar to copper transporters, including the high affinity Ctr3 copper transporter of *Saccharomyces cerevisiae* (S1 Fig). Each mutant had approximately 50% reduced RFP-fluorescence (intramacrophage fungal growth) and 60% reduced macrophage lysis compared to wild type (Fig 1A and 1B). Consistent with the RFP-based measurement of intracellular growth, CFU-based measurement of viable yeasts within macrophages in culture confirmed that the Ctr3-deficient mutant proliferated only 30% to 50% as well as wild type at 48 hours and 72 hours post-infection (Fig 1C). However, the *ctr3* mutant grew as well as the wild-type *CTR3* parent in liquid culture (Fig 1D). Complementation of the *ctr3* mutants with the wild-type *CTR3* gene restored intramacrophage proliferation (Fig 1A) and virulence in macrophages (Fig 1B) linking intramacrophage growth to Ctr3 function. We also found that Ctr3 is required for intracellular proliferation in another phylogenetically distinct *H*. *capsulatum* strain G186A, but the virulence defect observed in G186A yeasts was not as significant as that in G217B background strain (S2A Fig).

### Yeast growth requires Ctr3 under copper-limited conditions

**Fig 2:**
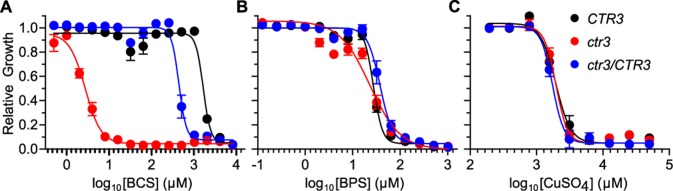
Ctr3 enables *H*. *capsulatum* growth in limited copper. Dose response curves for Ctr3-expressing (*CTR3*, black), Ctr3-deficient (*ctr3*, red), and the complemented (*ctr3/CTR3*, blue) strains grown in liquid culture with the copper-specific chelator BCS **(A)**, the iron-specific chelator BPS **(B)**, and with added CuSO_4_ **(C)**. Yeasts were grown in HMM at 37°C in the presence of a gradient of concentrations of BPS, BCS or CuSO_4_. Yeast growth was monitored daily by measurement of culture turbidity (optical density at 595 nm) and growth after 5 days normalized to growth of yeasts in the absence of chelators or CuSO_4_. Dose-response curves were generated by non-linear regression. IC_50_ values for growth in BCS were 1,661 μM, 2.8 μM, and 442 μM, for *CTR3*, *ctr3*, and *ctr3/CTR3*, respectively. Data represent the average relative growth ± standard deviation of biological replicates (n = 3).

Consistent with the function of Ctr3 homologs in other fungi, Ctr3 enables *H*. *capsulatum* acquisition of copper when copper is limited. Restriction of copper showed Ctr3-deficient yeasts are more sensitive to reduced copper availability; the IC_50_ of the copper chelator bathocuproine disulfonate (BCS) for the *ctr3* mutant is over 150-fold lower than that of the *CTR3* parent and *ctr3/CTR3* complemented strains (Fig 2A). A similar pattern was observed in the G186A strain background; lack of Ctr3 rendered yeasts approximately 10-fold more sensitive to BCS (S2B Fig). Supplementation of BCS-chelated media with excess copper, but neither zinc nor iron, restored the growth of the *ctr3* mutant demonstrating the specificity of the phenotype for copper (S3 Fig). Consistent with this, loss of Ctr3 function did not affect growth in iron restricted conditions (using the iron chelator bathophenanthroline disulfonate (BPS); Fig 2B), suggesting that Ctr3 plays no role in iron uptake (Fig 2B). In contrast to copper-chelation, loss of Ctr3 function does not affect *H*. *capsulatum* growth in high copper as both the *ctr3* mutant and *CTR3* parent strain grow equally well in media with millimolar concentrations of copper (Fig 2C).

**Fig 3:**
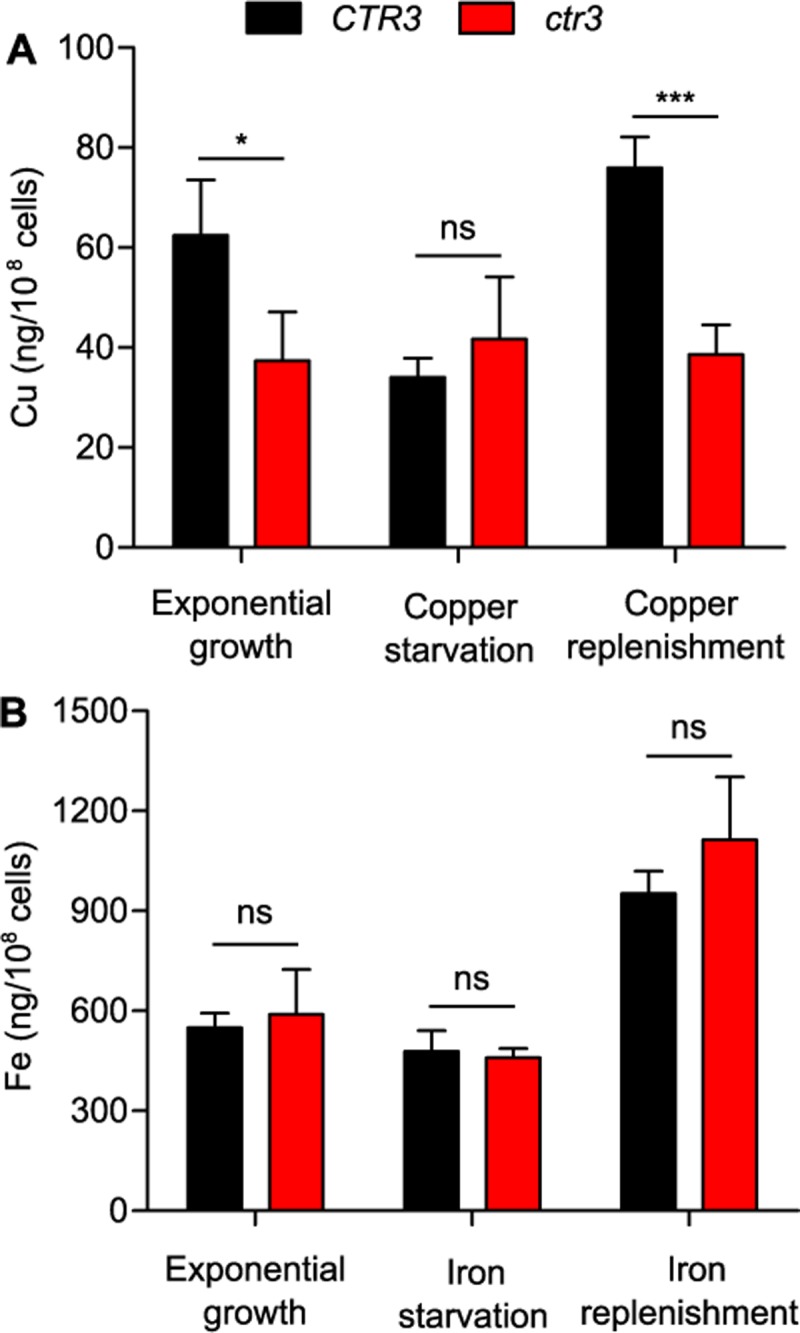
*Histoplasma* Ctr3 enables copper import. Copper (**A**) and iron (**B**) acquisition of Ctr3-expressing (*CTR3*, black) and Ctr3-deficient (*ctr3*, red) yeasts. Total cellular metal levels were measured by ICP-MS of yeasts in exponential growth in HMM (which contains 10 nM CuSO_4_ and 3 μM FeSO_4_) before and after metal depletion (treatment of yeasts for 24 hours with 2 mM BCS (**A**) or 16 μM BPS (**B**)) and after 3 hours with replenishment of copper and iron in the growth medium. Data represent the average level of copper or iron per 10^8^ yeasts ± standard deviations among biological replicates (n = 3). Asterisks represent significant differences in metal levels between Ctr3-expressing and Ctr3-deficient yeasts (* *P* < 0.05, *** *P* < 0.001, ns *P* > 0.05) as determined by two-tailed Student’s *t*-test.

To directly show Ctr3-dependent copper acquisition, we measured intracellular copper levels in yeasts by inductively-coupled plasma mass spectrometry (ICP-MS). Ctr3-deficient yeasts have lower overall copper levels compared to Ctr3-expressing yeasts during exponential growth in medium containing 10 nM copper (Fig 3A), and starvation of yeasts for copper reduces intracellular copper to baseline levels. Upon replenishment of copper, Ctr3-expressing but not Ctr3-deficient yeasts accumulate intracellular copper (Fig 3A). Both Ctr3-expressing and Ctr3-deficient yeasts have equivalent iron levels in exponential growth and after iron starvation and accumulate iron upon supplementation (Fig 3B), showing the specificity of Ctr3 for copper, but not iron acquisition. Together these data indicate that Ctr3 functions as a copper-specific importer to facilitate growth of yeasts when available copper is low.

### Copper limitation and differentiation into pathogenic yeasts both induce CTR3 expression

**Fig 4:**
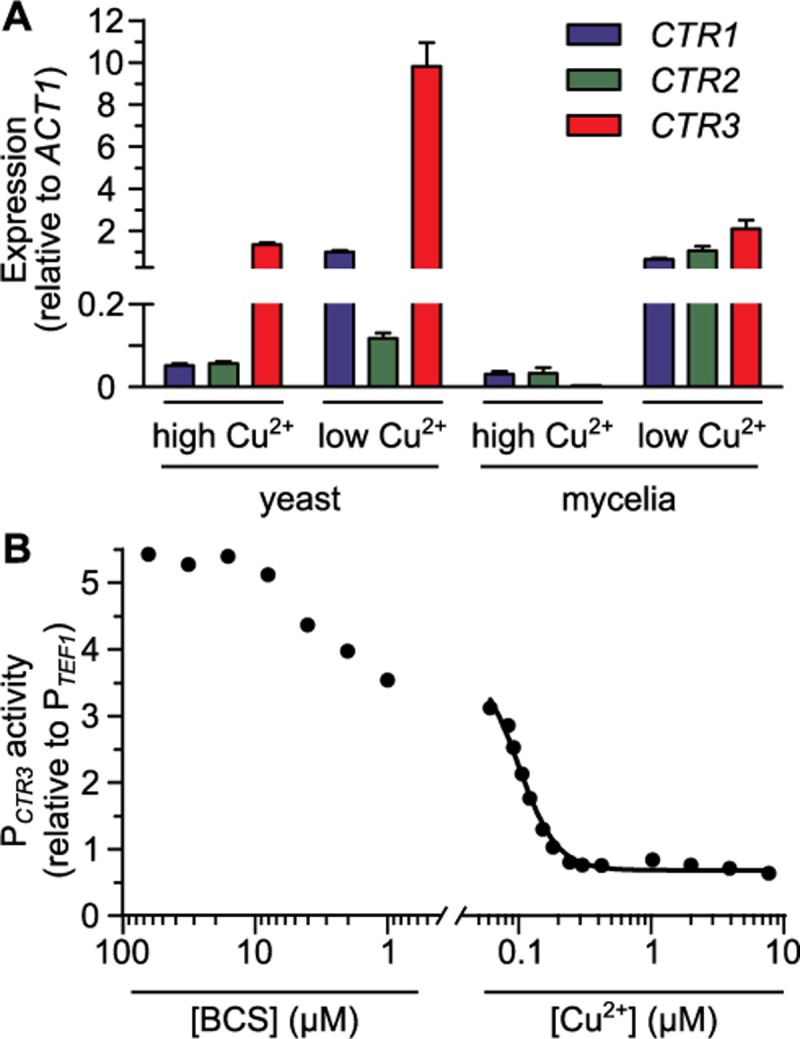
Copper limitation and differentiation into pathogenic yeast phase induce *CTR3* expression. Expression of *CTR* genes based on qRT-PCR **(A)** or a *CTR3* promoter fusion to *gfp* **(B)**. Expression was calculated relative to the constitutive expression of the *TEF1* gene. **(A)** *H*. *capsulatum* cells in the host-infecting form (yeasts, 37°C) and environmental form (mycelia, 25°C) were grown in 3M media with low (10 nM) or high (10 μM) CuSO_4_ added and the *CTR1* (blue), *CTR2* (green), and *CTR3* (red) transcript levels were quantified by qRT-PCR and calculated relative to *ACT1* transcript levels. Data represent the average expression ± standard deviation of results from biological replicates (n = 3). **(B)** *CTR3* promoter activity (P_*CTR3*_) of yeasts grown in 3M medium with different copper concentrations was measured by fluorescence and normalized to the fluorescence of a strain with a *TEF1* promoter-*gfp* fusion (P_*TEF1*_). Fluorescence intensity was measured under different CuSO_4_ concentrations with concentrations lower than 60 nM (the trace amount present in 3M medium) achieved by addition of the copper chelator BCS. A non-linear regression standard curve (black line) was fit to the data with known copper concentrations. Data represent average *CTR3* promoter activity of replicates (n = 3).

The Ctr3 requirement for yeast growth in low copper suggests *CTR3* expression may be regulated by copper concentrations. Bioinformatic analysis of the *H*. *capsulatum* genome identified two additional putative copper transporters which were designated Ctr1 and Ctr2 (S1 Fig). Examination of *CTR1*, *CTR2*, and *CTR3* gene expression by qRT-PCR showed that low copper (10 nM) significantly increased mRNA levels of all three *CTR* genes compared to high copper (10 μM) conditions; *CTR1*, *CTR2*, and *CTR3* were all induced in low copper media compared to high copper media, regardless of whether cells were grown as yeasts or mycelia (Fig 4A). Interestingly, *CTR3* had the highest overall expression of the *CTR* genes. In mycelia, *CTR3* expression was induced by low copper and was expressed at similar levels to that of *CTR1* and *CTR2*. However, in yeast cells, the expression of *CTR3* in high copper was 2.5-fold higher than that of *CTR1* and *CTR2* (Fig 4A) and *CTR3* expression was further induced nearly 10-fold when yeasts were grown in low copper (Fig 4A). These data indicate that while expression of *CTR1*, *CTR2*, and *CTR3* are all induced by low available copper, differentiation of *H*. *capsulatum* cells into pathogenic yeasts establishes an overall higher baseline of expression.

Since copper regulates *CTR3* expression, we created a green-fluorescent protein (*gfp*) transcriptional fusion to the *H*. *capsulatum CTR3* promoter as a fluorescent indicator of copper availability. *CTR3* promoter activity, as indicated by GFP fluorescence of yeast cells, was measured by microscopy after growth in liquid culture and normalized to the fluorescence of an isogenic strain in which *gfp* expression was controlled by the constitutive *H*. *capsulatum TEF1* promoter. Consistent with the transcriptional analysis, decreasing copper concentrations increased the *CTR3* promoter activity, and addition of BCS further increased the reporter GFP-fluorescence to levels at least 5-fold greater than expression in high copper (Fig 4B). Conversely, addition of copper greatly decreases but does not eliminate *CTR3* promoter activity. The *CTR3* promoter responds to changes in copper concentrations but is not affected by changes in iron or zinc (S4 Fig). In addition, the *CTR3* promoter activity is not affected by reactive oxygen stress (S5A Fig) or changes in pH (S5B Fig), two physiologically-relevant environmental changes encountered by Histoplasma in the phagosome. These data indicate the specificity of the *CTR3* promoter regulation for available copper.

To provide quantitative estimates of phagosomal copper levels, the fluorescence of the *gfp* reporter strains was measured in a gradient of copper concentrations. Analysis of media by inductively coupled plasma mass spectrometry (ICP-MS) showed that media without any metal addition had 60 nM trace copper. To reduce copper concentrations below 60 nM necessitated culture of cells in increasing concentrations of BCS. The dose response-data for increasing copper was used to generate a curve of the *CTR3*-promoter (P_*CTR3*_)-controlled GFP fluorescence in 60 nM to 10 μM copper, which showed that *CTR3* promoter activity decreased to baseline levels at concentrations above 240 nM copper (Fig 4B). Maximal *CTR3* promoter activity reached a plateau of approximately 5-fold higher relative expression in media containing at least 8 μM of BCS (Fig 4B). These data show that the *CTR3* promoter is regulated by copper and the *CTR3* promoter-dependent fluorescence of *gfp* reporter yeasts provides an estimate of the available copper in *H*. *capsulatum*’s environment.

### *H*. *capsulatum* virulence in vivo requires Ctr3 during the adaptive immune response

**Fig 5:**
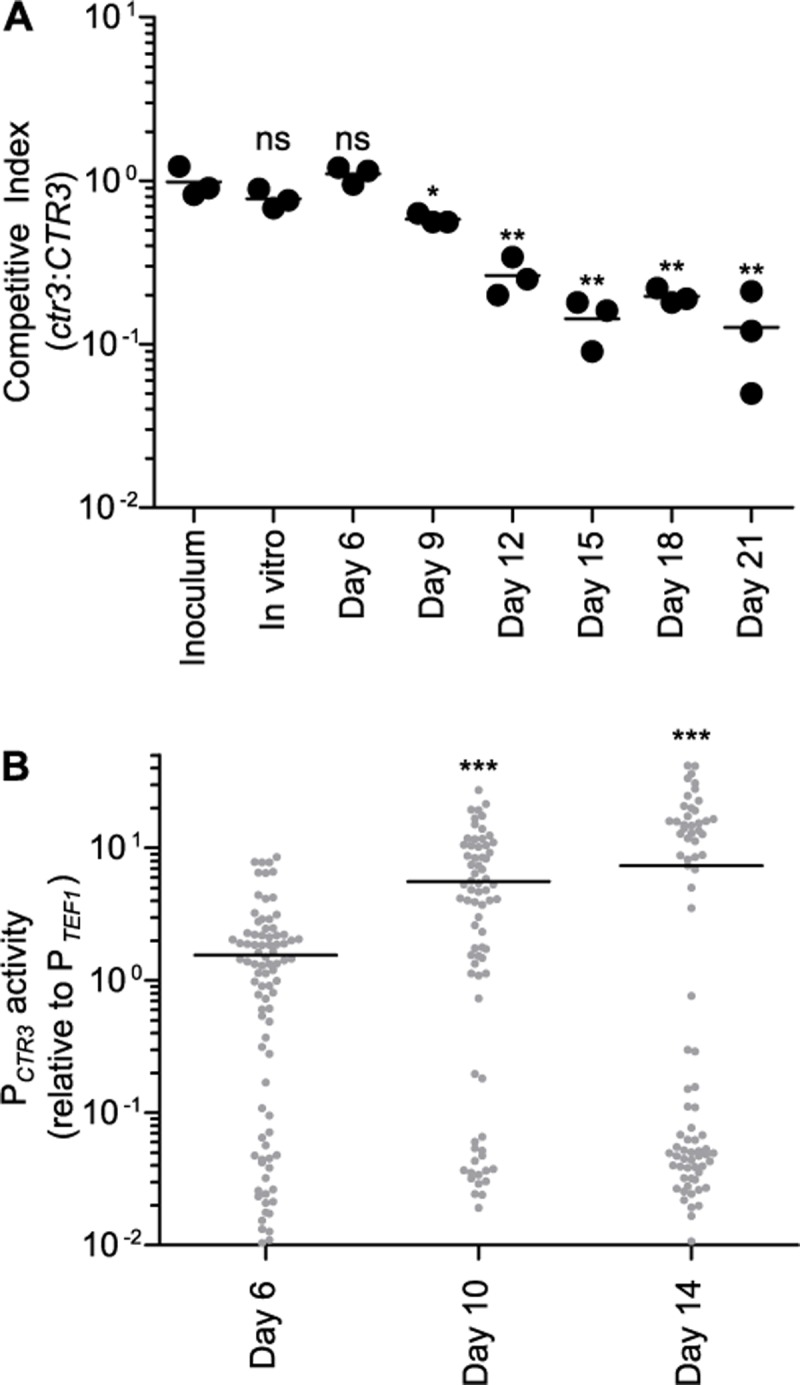
*H*. *capsulatum* virulence in vivo requires Ctr3 during the adaptive immune response. Proliferation of *H*. *capsulatum* yeasts **(A)** and *CTR3* promoter activity **(B)** in vivo following respiratory infection of mice. **(A)** The relative fitness (competitive index) of Ctr3-deficient (*ctr3*) compared to Ctr3-producing (*CTR3*) yeasts was determined by co-infecting mice intranasally with equal amounts of *CTR3* (non fluorescent) and *ctr3* (GFP-fluorescent) yeasts (1x10^4^ each). Fungal burdens were determined at 6, 9, 12, 15, 18, and 21 days post-infection by harvesting lungs, plating lung homogenates on solid medium, and enumerating fluorescent and non-fluorescent CFU. Data points represent the ratio of GFP-fluorescent to GFP-negative CFU at each time point (n = 3 mice) with the average ratio indicated (horizontal bar). Asterisks indicate significant (* *P* < 0.05, ** *P* < 0.01) differences compared to the ratio of the inoculum as determined by one-tailed Student’s *t*-test. **(B)** *CTR3* promoter activity in vivo was determined by infecting mice intranasally with wild-type yeasts expressing either the *CTR3* promoter-*gfp* fusion (P_*CTR3*_) or a *TEF1* promoter-*gfp* fusion (P_*TEF1*_) and collecting lung tissue at 6, 10, and 14 days post infection. Lung cells were lysed to release intracellular yeasts and the fluorescence of yeasts quantified by microscopy. Fluorescence of individual yeasts with the *CTR3* promoter-*gfp* fusion was normalized to the average fluorescence of yeast with the *TEF1* promoter-*gfp* fusion. Data points represent the *CTR3* promoter activity of individual yeasts and bars represent the average results from replicate infections (n = 3). For each infection, at least 60 yeasts were analyzed. Asterisks indicate significant (*** *P* < 0.001) differences compared to the *CTR3* promoter activity at day 6 post-infection as determined by one-tailed Student’s *t-*test.

To determine the role of Ctr3 in *H*. *capsulatum* virulence, Ctr3-producing and Ctr3-deficient yeasts were tested in a murine model of pulmonary histoplasmosis. Respiratory infections of mice were established using a mixed inoculum of the wild-type and *ctr3* mutant strains and the fungal burden in lungs determined over time. To enable measurement of the relative fitness of the Ctr3-deficient *ctr3* strain, the *ctr3* mutant strain was marked with constitutive GFP-fluorescence, and the viable colony forming units (cfu) of wild-type versus mutant strains were differentiated by colony fluorescence. At day 6 post-infection, a time point before significant adaptive immune responses, the *ctr3* mutant showed equivalent lung infection as wild type (Fig 5A). However, after the peak of the adaptive immune response (day 9–21 post-infection), the *ctr3* mutant was less fit compared to the co-infecting wild-type strain. Complementation of the *ctr3* mutant with the *CTR3* gene restored the virulence of the mutant (S6 Fig) indicating the loss of fitness was due to loss of Ctr3 function. These data show that Ctr3 is required for full virulence, specifically at time points following activation of cell-mediated immunity. The requirement for Ctr3 function in *H*. *capsulatum* growth both in limited copper (Fig 2A) and for pathogenesis during adaptive immune response stages suggests that copper becomes limiting in the phagosome of phagocytes during adaptive immunity.

To probe the intraphagosomal copper concentration during *H*. *capsulatum* infection, we used the copper-regulated *CTR3* promoter-*gfp* fusion to measure the *CTR3* promoter activity in vivo. Following respiratory infections in mice, *H*. *capsulatum* yeasts were collected from lung tissue and the fluorescence of the *CTR3* promoter-*gfp* yeasts measured. Consistent with the equivalent fitness of the wild type and the Ctr3-deficient strain during innate immunity (Fig 5A), the *CTR3* promoter activity remained low at 6 days post-infection (Fig 5B). Comparing the yeast GFP fluorescence to the copper concentration dose-response curve for the CTR3 promoter (Fig 4B) estimates the copper concentration *H*. *capsulatum* encounters at day 6 post-infection is approximately 100 nM, a concentration sufficient to allow yeast growth without Ctr3 function (Fig 2A). However, the *CTR3* promoter activity at day 10 and day 14 post-infection was 3- to 5-fold higher than that at day 6 post-infection indicating less available copper in the *H*. *capsulatum*-containing phagosome at these time points (Fig 5B). At day 14 post-infection, the fluorescence distribution appears bimodal. The low fluorescent yeasts may reflect a sub-population that is inhibited for growth due to macrophage activation or that not all phagocytes have equivalent changes in phagosomal copper. The average *CTR3* promoter activity measured in yeast in vivo at day 10 and 14 (including the low-fluorescent population) was similar to growth in liquid medium containing at least 64 μM BCS, a concentration which induces the *CTR3* promoter (Fig 4B) and at which the *ctr3* mutant cannot grow (Fig 2A). Together these data suggest that phagosomal copper becomes significantly limited in phagocytes during the adaptive immune response.

### Cytokine activation of macrophages decreases phagosomal copper levels

**Fig 6:**
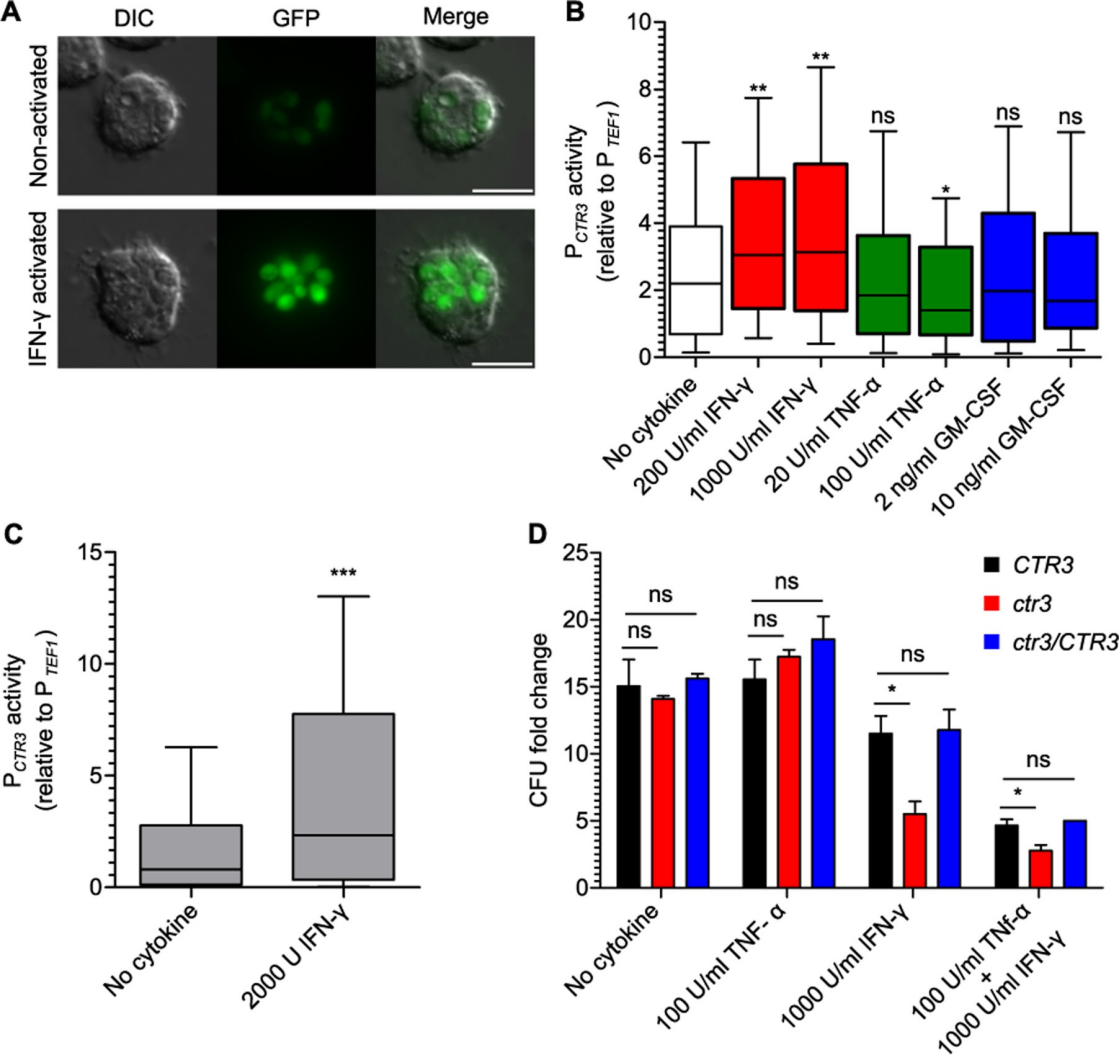
Activation of macrophages decreases phagosomal copper availability and impairs proliferation of Ctr3-deficient yeasts. Phagosome copper dynamics following cytokine activation of macrophages **(A, B, and D)** or in vivo administration of IFN-γ **(C)**. **(A)** Representative differential interference contrast (DIC) and fluorescence (GFP) images of intracellular *H*. *capsulatum* yeasts with the *CTR3* promoter-*gfp* fusion in non-activated BMDMs (top panel) or BMDMs activated with 1000 U/mL IFN-γ (bottom panel). Scale bar represents 10 μm. **(B)** BMDMs were treated with IFN-γ, TNF-α, or GM-CSF and infected with *H*. *capsulatum* yeast (MOI 1:2) with *TEF1* (P_*TEF1*_) or *CTR3* (P_*CTR3*_) promoter-*gfp* fusions. After 48 hours *CTR3* promoter activity of intramacrophage yeasts was determined by lysis of macrophages, recovery of yeasts, and measurement of GFP-fluorescence by microscopy (n > 100 yeasts for each sample) and normalized to the average *TEF1* promoter activity. **(C)** Activation of the *CTR3* promoter in vivo following IFN-γ treatment was determined by infecting mice intranasally with wild-type *H*. *capsulatum* yeasts with the P_*TEF1*_ or P_*CTR3*_ promoter *gfp* fusions. 2000 U of IFN-γ was delivered to the lungs at day 4 and 5 post-infection, and at 6 days post-infection, lungs were harvested, lung cells lysed to release intracellular yeasts, and the GFP fluorescence quantified by microscopy (n > 100 yeasts). **(B-C)** Data represent the *CTR3* promoter activity among biological replicates (n = 3) after normalization to *TEF1* promoter activity. Box plot shows upper and lower quartiles with the median value (horizontal bar). Vertical bars indicate 10% to 90% of the data distribution. Asterisks indicate significant differences compared to non-activated macrophages (* *P* < 0.05, ** *P* < 0.01, *** *P* < 0.001). **(D)** BMDMs were activated with IFN-γ, TNF-α, or both cytokines and infected with Ctr3-expressing (*CTR3*, black), Ctr3-deficient (*ctr3*, red) and complemented (*ctr3/CTR3*, blue) *H*. *capsulatum* yeasts (MOI 1:2). After 48 hours, intracellular yeasts were recovered and enumerated by plating for CFU. Data represent the average fold change ± standard deviation compared to yeasts at the start of the assay among biological replicate infections (n = 3). Asterisks indicate significant differences compared to the intracellular proliferation of *CTR3* yeasts (* *P* < 0.05).

As one of the central features of the adaptive immune response involves cytokine activation of phagocytes, we tested which cytokines induce phagosomal copper restriction in *H*. *capsulatum*-infected macrophages. For these experiments, the *H*. *capsulatum CTR3* promoter-regulated GFP fluorescence was used to indicate the levels of available copper within the macrophage phagosome. *H*. *capsulatum* yeasts within the phagosome of non-activated bone marrow-derived macrophages (BMDMs) expressed GFP at moderate levels (Fig 6A). Quantification of the *CTR3*-driven GFP fluorescence and correlation with the in vitro-derived dose-response data (Fig 4B) estimates the phagosomes of unactivated macrophages is around 80 nM. Treatment of *H*. *capsulatum*-infected BMDMs with IFN-γ increased *CTR3* promoter activity in a dose-dependent manner indicating phagocyte activation with IFN-γ stimulates restriction of phagosomal copper availability (Fig 6A and 6B). However, treatment with TNF-α or GM-CSF did not significantly increase *CTR3* promoter activity, suggesting these cytokines do not significantly influence phagocyte phagosomal copper concentrations (Fig 6B).

IFN-γ-induced phagosomal copper restriction also occurs in vivo. At day 6 post-infection, the *CTR3* promoter activity is normally low (Fig 5B). However, administration of IFN-γ to mice increased the *CTR3* promoter activity of *H*. *capsulatum* yeasts (Fig 6C); the average fluorescence was at least two-fold higher with just two IFN-γ treatments indicating that IFN-γ is sufficient to induce copper restriction in vivo. Copper concentration estimation using the dose-response curve (Fig 4B) indicates that this IFN-γ treatment causes copper concentration to decrease from 100 nM to well below 60 nM.

We used the growth of Ctr3-deficient yeasts as an alternate indicator of cytokine-induced changes to phagosomal copper availability. Intramacrophage growth of Ctr3-deficient yeasts, which are sensitive to low copper, was compared to that of wild-type *H*. *capsulatum* yeasts. Without cytokine treatment, Ctr3-deficient yeasts proliferate equally as well in BMDMs as Ctr3-expressing yeasts (Fig 6D). This indicates copper is not limited in the phagosomes of these macrophages and is consistent with the *CTR3* promoter activity measurements (Fig 6A and 6B). Treatment of BMDMs with IFN-γ, but not TNF-α, restricted the growth of Ctr3-deficient *H*. *capsulatum* yeasts (Fig 6D), demonstrating that IFN-γ triggers restriction of available copper in the phagosome to levels which impair the growth of Ctr3-deficient yeasts. These results are consistent with the *CTR3* promoter activity data in IFN-γ-treated macrophages (Fig 6A and 6B) indicating that IFN-γ activation of macrophages changes the available phagosomal copper from high to low concentrations (significantly less than 60 nM).

For quantifying *CTR3* promoter activity, GFP fluorescence driven by the *CTR3* promoter was normalized to fluorescence of intracellular yeasts with a GFP-promoter fusion to the *TEF1* promoter to control for any changes in global gene expression due to the state of intracellular yeast cells. The activity of the *TEF1* promoter is not affected by copper levels (S7A Fig) or by residence within unactivated or activated macrophages in culture (S7A Fig). Furthermore, normalization of the GFP fluorescence driven by the *CTR3* promoter to a different housekeeping gene (*H2B*) promoter fusion showed a similar increase of the *CTR3* promoter in intramacrophage yeasts before and after activation (S7B Fig). Finally, GFP-fluorescence driven by the *CTR3* promoter was normalized to RFP-fluorescence driven by the *TEF1* promoter within the same yeast cells. This also showed the same induction of the *CTR3* promoter in activated BMDMs (S7C Fig). These data indicate that *TEF1* promoter activity serves as an accurate normalization factor to account for global transcription variation due to intracellular residence of yeasts.

### Available intraphagosomal copper concentration differs among primary macrophages

**Fig 7:**
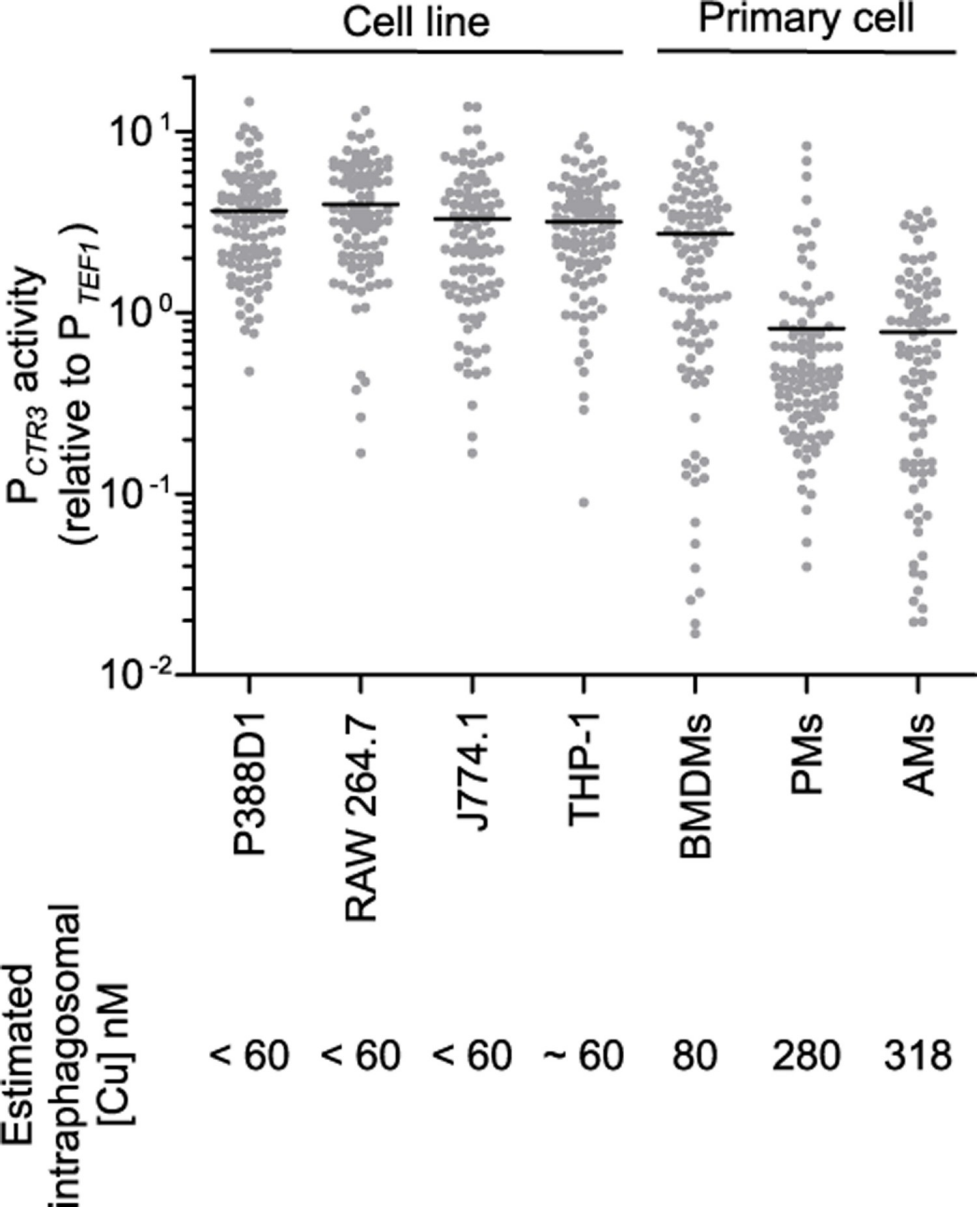
Phagosomal copper concentration differs among primary macrophages and macrophage cell lines. Common macrophage cell lines (P388D1, RAW264.7, J774.1, and PMA-differentiated THP-1 cells) and unactivated primary cells (bone marrow derived macrophages (BMDMs), peritoneal macrophages (PMs), and alveolar macrophages (AMs)) were infected with *H*. *capsulatum* yeasts (MOI 1:2) with *TEF1* or *CTR3* promoter-*gfp* fusions for 48 hours. Macrophages were lysed, intracellular yeasts recovered, and the GFP fluorescence of individual yeast was quantified by microscopy (n > 100 yeasts for each sample). *CTR3* promoter activity as indicated by the *CTR3* promoter-*gfp* fusion was normalized to the average fluorescence of the *TEF1* promoter-*gfp* fusion. Horizontal bars represent the average *CTR3* promoter activity.

Surveying primary murine phagocytes as well as common macrophage cell lines showed that *CTR3* promoter activity of intracellular *H*. *capsulatum* yeasts was high in cultured macrophage cell lines, indicating significantly restricted phagosomal copper even without cytokine treatment in these cells (Fig 7). A similar pattern among cell lines was also observed when the *H2B* promoter was used for normalization (S8A Fig) or when the *CTR3*-driven GFP fluorescence was normalized to the *TEF1* promoter activity within the same cells (S8B Fig). Among primary cells, resident peritoneal macrophages and alveolar macrophages had high phagosomal copper concentrations (Fig 7) estimated at 280 nM and 320 nM, based on the copper dose-response curve for the *CTR3* promoter (Fig 4B). This is consistent with the equivalent in vivo proliferation of the Ctr3-deficient and Ctr3-expressing yeasts when *H*. *capsulatum* yeasts are primarily present in alveolar macrophages before phagocytes are activated.

## Discussion

In order to establish infections and proliferate in macrophages, *H*. *capsulatum* yeasts must acquire essential metals within the phagosomal environment. *H*. *capsulatum* secretes siderophores and expresses zinc transporters to combat host limitation of iron and zinc, respectively. In this study, we demonstrate that growth in macrophages also imposes challenges on yeasts to maintain copper homeostasis. Specifically, *H*. *capsulatum* yeasts rely on the Ctr3 copper transporter to acquire sufficient copper when copper becomes limiting, both in liquid culture and within macrophages. Besides Ctr3, the *H*. *capsulatum* genome encodes two additional putative copper transporters (Ctr1 and Ctr2). However, Ctr1 and Ctr2 are not simply redundant with Ctr3; Ctr1 and Ctr2 are not as highly expressed as Ctr3, and they are not sufficient for copper acquisition when phagosomal copper levels become severely limited. Thus, Ctr3 is the primary transporter involved in copper acquisition as part of *H*. *capsulatum*’s pathogenesis program.

Two aspects related to *H*. *capsulatum* pathogenesis contribute to Ctr3 expression. First, differentiation of *H*. *capsulatum* into pathogenic yeasts induces Ctr3 expression independent of copper levels, consistent with a virulence role facilitating *H*. *capsulatum* infection of macrophages and persisting within a copper-limited environment. Second, restriction of available copper further increases Ctr3 expression above the level set by yeast-phase differentiation. In support of this dual regulation of Ctr3 transcript levels, the Ctr3 promoter contains putative binding sites for two transcription factors, Ryp1 and Mac1, which have been implicated in yeast-phase gene regulation and fungal transcriptional responses to copper concentration, respectively [17,29,30]. Furthermore, ChIP-chip analysis showed that two yeast-phase transcriptional regulators, Ryp1 and Ryp4, preferentially interact with the *CTR3* locus in the yeast phase compared to mycelia [29]. While we can not rule out the possibility that other copper-independent features of macrophage infection do not impact the *CTR3* promoter activity, the data suggest that yeast-phase expression of *CTR3* is regulated primarily by available copper. Other physiologically-relevant conditions encountered by yeasts within the phagosome (i.e., iron and zinc concentrations, reactive oxygen, and pH changes) do not influence the *CTR3* promoter. Consistent with our results, *CTR3* is part of a copper-responsive regulon in a microarray-based study of copper-regulated genes [31], although in this study only yeast responses were examined. *H*. *capsulatum* strain differences in *CTR3* expression are due to trans-acting factors [32], likely from variations in either Ryp or Mac1 production or activity among strains. Together, these data are consistent with the model that yeast phase differentiation primes *H*. *capsulatum* cells for pathogenesis by inducing basal Ctr3 expression and the level of Ctr3 production is further tuned to the precise level of copper availability in the phagosome.

Technical challenges in direct measurement of phagosomal copper required the use of surrogate indicators of copper availability for intracellular *H*. *capsulatum*. Using a transcriptional *gfp* fusion as a semi-quantitative reporter of available copper, we determined that during innate immune stages, *H*. *capsulatum* resides within a phagosomal environment with copper concentrations above 100 nM. Consistent with this, *CTR3* transcription is lowest for yeast within alveolar macrophages (Fig 7). At 10 days post-infection, which coincides with the onset of adaptive immune responses, copper becomes restricted. Both in vitro and in vivo, IFN-γ is sufficient to trigger copper restriction. Comparison of in vivo *CTR3* transcription to a standard curve generated in vitro estimates phagosomal copper concentrations become significantly lower than 60 nM. As *H*. *capsulatum* yeasts are found almost exclusively within phagocytes during mammalian infection [33], these levels reflect copper concentration within the phagosomal environment at these two points of infection. We note that these copper concentrations are inferred using regulation of the *CTR3* promoter, which assumes differential expression is not influenced by copper-independent changes during infection. However, our conclusions about *CTR3* transcription reflecting copper dynamics are completely supported by the differential growth of Ctr3-deficient and Ctr3-expressing *H*. *capsulatum* strains as a second indicator of copper availability within the phagosome; Ctr3-mediated copper transport is required for *H*. *capsulatum* yeast proliferation in vivo at 9 days post-infection but not before. While higher intraphagosomal copper concentration can be microbicidal to some intracellular pathogens, *H*. *capsulatum* can tolerate high copper (up to mM levels) and thus copper toxicity mechanisms of immune defense are ineffective.

As a nearly exclusive intracellular pathogen, *H*. *capsulatum* responses to copper levels provide unique insights into the dynamics of the phagosomal environment. Like *H*. *capsulatum*, *C*. *neoformans* (*grubii*) yeasts up-regulate transcription of copper transporters (Ctr4 and Ctr1 which are homologs of *H*. *capsulatum* Ctr3 and Ctr1, respectively) in response to copper restriction [30,34,35]. For *C*. *neoformans*, these transcriptional responses vary by tissue with *CTR4* promoter activity increasing in the CNS environment but not in the lung environment, suggesting limited copper in the CNS, but not the lung [17,22,35]. These results are corroborated by the reciprocal expression profile of *C*. *neoformans* copper-binding metallothioneins (Cmt1 and Cmt2) which are induced by high copper concentrations; *C*. *neoformans* yeasts in the lung have elevated Cmt1 and Cmt2 expression but not in the brain [17,22]. Multiple functional studies with mutants of *C*. *neoformans* support the transcriptional profiles since Ctr4- and Ctr1-deficient *C*. *neoformans* yeasts, which are unable to grow in limited copper [23,30], are impaired in CNS but not lung infection [22,23,30,36]. In contrast, *C*. *neoformans* mutants lacking the Cmt1 and Cmt2 metallothioneins are attenuated in lung infection. Together these studies indicate copper concentrations in the lungs during *C*. *neoformans* infection are sufficiently high to not require the Ctr1 and Ctr4 transporters. Initially, these findings appear to contrast with those we observe with *H*. *capsulatum* yeasts. However, while *H*. *capsulatum* yeasts are nearly exclusively intracellular during infection [33], *C*. *neoformans* has multiple mechanisms to avoid long-term residence within macrophages (e.g., formation of phagocytosis-resistant titan cells [37], production of the anti-phagocytic capsule [38], secretion of anti-phagocytic protein App1 [39], and vomocytosis [40]). Thus, *C*. *neoformans* infection studies indicating the lung is not copper limiting likely include the general extracellular environment, whereas *H*. *capsulatum* yeasts indicate phagosome-specific copper concentrations. Indeed, *C*. *neoformans* infection of RAW264.7 or J774.1 macrophages in vitro show up-regulation of *CTR4* but not *CMT1* expression in intracellular yeasts consistent with our data showing the phagosome in these macrophage cell lines have low available copper [23,35]. In addition, Ding et al. found that expression of the mammalian phagosomal copper transporter ATP7A decreased in bronchoalveolar lavage cells at day 14 following *Cryptococcus* infection consistent with reduced transport of copper into the phagosome during adaptive immunity [17]. These findings and our results with *Histoplasma* establish a general model that while the extracellular lung environment has ample copper, the phagosome of lung phagocytes becomes copper limiting, particularly following IFN-γ activation.

Copper restriction as a mechanism to control fungal pathogens contrasts with copper toxicity as a means to control bacterial pathogens. Phagosomes of macrophages infected with the intracellular bacterial pathogen *M*. *tuberculosis* have approximately 400 μM Cu after 1 hour which decreases to 20 μM after 24 hours [41]. Despite this decrease in copper, 20 μM is still a considerably high amount of copper. Consistent with elevated copper levels in the *M*. *tuberculosis*-containing phagosome, *M*. *tuberculosis* bacteria which have lost the outer membrane copper export protein have reduced tolerance to copper and reduced virulence compared to wild type [19]. Similarly, *M*. *tuberculosis* mutants in the RicR regulon are inhibited by high copper (> 60 μM) in vitro and are attenuated in vivo [42]. Loss of the CsoR regulon improves copper resistance of *M*. *tuberculosis* enabling full virulence during early stages of infection. These data indicate that the *M*. *tuberculosis*-containing phagosome contains relatively high concentrations of copper. Supporting this, treatment of macrophages with LPS or IFN-γ increases the host copper transporting protein ATP7A on the phagosome membrane [43]. Even though these studies lack direct measurement of intraphagosomal copper concentrations, they are consistent with findings of elevated copper within latex bead-containing phagosomes and a requirement for ATP7A for phagocyte killing of *Escherichia coli* [43]. In contrast, 14 days following *C*. *neoformans* pulmonary infection (a time point consistent with IFN-γ production) alveolar macrophages have decreased ATP7A levels [17]. These differences and our data with intramacrophage *H*. *capsulatum* yeasts suggest that macrophages may differentiate between bacterial and fungal pathogens and employ copper toxicity or copper limitation, respectively, in their attempts to limit replication of these two classes of pathogens.

With the involvement of adaptive immunity, host utilization of copper for control of fungi switches from copper toxicity to copper restriction. *Aspergillus fumigatus* conidia infection of alveolar macrophages increases ATP7A expression consistent with elevation of phagosomal copper [18] as an initial phagocyte response. *A*. *fumigatus* conidia lacking the AceA transcription factor are less tolerant of high copper and accordingly are less virulent in vivo [18]. Aspergillus cells lacking two Ctr transporters homologous to the fungal Ctr3/Ctr4 and Ctr1 proteins are unable to grow in low copper but lack any virulence defects in vivo [44]. These data indicate that copper toxicity is the primary host defense initially employed against fungal cells (i.e., during the innate immune response). In contrast to *H*. *capsulatum* yeasts, innate immune mechanisms are sufficient for control of *A*. *fumigatus* infections and cells that escape initial clearance by phagocytes grow as extracellular hyphae. *H*. *capsulatum* yeasts, on the other hand, are not controlled by innate immunity and are primarily intracellular. Instead restriction of *H*. *capsulatum*, as well as *C*. *neoformans*, requires activation of phagocytes by the adaptive immune system. Our data shows that IFN-γ is key to the switch of macrophage phagosomes from a high copper environment to a copper-limited environment, and it explains, in part, how adaptive immunity contributes to the control of intracellular primary pathogens.

## Materials and methods

### *H*. *capsulatum* strains and growth

*H*. *capsulatum* strains used in this study are listed in the S1 Table and were derived from the G217B and G186A clinical isolates. *H*. *capsulatum* yeasts were grown in *H*. *capsulatum*-macrophage medium (HMM, which contains 10 nM CuSO_4_) or in 3M media [45] without added copper for metal supplementation tests with FeSO_4_, ZnSO_4_, or CuSO_4_ as appropriate. For growth of uracil auxotrophs, HMM was supplemented with 100 μg/ml uracil. Yeasts were grown with continuous shaking (200 rpm) at 37°C and mycelia cultures at 25°C. Cultures were grown to exponential phase for use in infection studies. For dose-response tests with chelators and metals, yeasts were grown at 37°C in microtiter plates with twice-daily agitation [46]. For growth on solid medium, HMM was solidified with 0.6% agarose and supplemented with 25 μM FeSO_4_.

Growth of G217B derived strains in liquid culture was quantified by measurement of culture turbidity (optical density at 595 nm) or enumerating viable CFU by plating dilutions on solid HMM. Growth of G186A derived strains was determined by resazurin-based yeast metabolic assay [46]. Briefly, 100 μM resazurin was added to the yeast culture at 37°C and resorufin fluorescence (530 nm excitation, 590 nm emission) was measured over 90 minutes.

### Macrophage cell culture

LacZ-expressing P388D1 cell line was created from mouse cell line P388D1 (ATCC CCL-46, [29]). LacZ-expressing P388D1 RAW264.7 (ATCC TIB-71) and J774.1 (ATCC TIB-67) macrophage cell lines were maintained in Ham’s F-12 medium supplemented with 10% fetal bovine serum (FBS, Atlanta Biologicals). L929 cells (ATCC CCL-1) were maintained in Dulbecco’s modified Eagle medium (DMEM) supplemented with 10% FBS. THP-1 cells (ATCC TIB-202) were maintained in RPMI-1640 medium supplemented with 10% (FBS) and were differentiated in 10 ng/ml phorbol 12-myristate 13-acetate (PMA) for 48 h before use. All cell lines were cultured at 37°C in 5% CO2/95% air. For infection experiments, macrophage cell lines were co-cultured with yeasts in Ham’s F-12 medium supplemented with 10% FBS.

Peritoneal macrophages were obtained from wild-type C57BL/6 mice by peritoneal lavage with phosphate-buffered saline (PBS). For elicitation of macrophages, peritoneal injection of 3% protease peptone was performed 4 days prior to lavage. Bone marrow cells were isolated from femurs of C57BL/6 mice (Charles River) and differentiated by culturing in Dulbecco’s modified Eagle medium (DMEM) supplemented with 30% L929 cell culture supernatant for 7 days to obtain bone marrow derived macrophages (BMDMs). Non-adherent cells were removed from plastic dishes by washing with PBS. Alveolar macrophages were obtained from C57BL/6 mice by bronchoalveolar lavage (BAL) with PBS. All primary cells were cultured in DMEM at 37°C in 5% CO2/95% air.

### Mutagenesis and isolation of *H*. *capsulatum* mutants with attenuated intramacrophage growth

*H*. *capsulatum* strain OSU233 was used as the genetic background for insertional mutagenesis. OSU233 was constructed by *A*. *tumefaciens*-mediated transformation of *H*. *capsulatum* yeasts [47] with plasmid pQS01 which contains the *apt3* gene for selection (G418-resistance) and the tdTomato red-fluorescent protein transgene under control of the *H*. *capsulatum TEF1* constitutive promoter. OSU233 yeasts were mutagenized by *A*. *tumefaciens*-mediated transformation [48] using strain LBA1100 harboring plasmid pBHt2 [26]. Bacteria and yeasts were co-cultured for 40 hours on solid *Agrobacterium* induction medium containing 0.1 mM acetosyringone at 25°C. Cells were then transferred to HMM medium containing 100 μg/ml uracil, 100 μg/ml hygromycin to select for *H*. *capsulatum* transformants, and 10 μg/ml tetracycline to counter select *A*. *tumefaciens*. Plates were incubated at 37°C for 10–12 days until transformants appeared. Individual transformants were picked into liquid HMM with 100 μg/ml uracil in wells of a 96-well microtiter plate and incubated at 37°C for 5 days.

Monolayers of P388D1 lacZ-expressing macrophage cells in 96-well microtiter plates [28] were then inoculated with mutant yeasts at a multiplicity of infection (MOI) of 1:1 (yeasts:macrophages). Intramacrophage growth of yeasts was monitored daily by measuring RFP fluorescence (530 nm excitation, 590 nm emission) with a Synergy 2 microplate reader (Biotek). After 7 days, surviving macrophages were quantified by removal of culture media from the infected macrophages, lysis of the remaining macrophages with 0.1% Triton X-100, addition of 1 mg/mL o-nitrophenyl-β-D-galactopyranoside (ONPG), and determination of the β-galactosidase activity (absorbance at 420 nm with correction at 600 nm). Mutants with at least 30% reduction in intramacrophage growth or in lysis of the macrophages were retained as candidate attenuated strains.

### Mapping of *H*. *capsulatum* T-DNA insertional mutants

The location of the T-DNA insertion in individual mutants was determined by thermal asymmetric interlaced PCR (TAIL-PCR; [49]). 100 ng of genomic DNA was used as the template for primary PCR, with a T-DNA left or right border-specific primer (LB11 or RB9) and one of four semi-random primers (LAD1-4). The primary PCR reaction was diluted 500-fold and used as the template for the secondary PCR with nested left- or right-border primers (LB12 or RB10) and the AC1 primer. PCR products were sequenced and aligned to the *H*. *capsulatum* genome sequence. T-DNA insertion at the *CTR3* locus was confirmed by PCR and sequencing using *CTR3*-specific primers in conjunction with LB11 and RB9. Primer sequences are listed in the Key Resource Table.

### Complementation of the *ctr3* mutation

A 1.7 kb fragment consisting of the wild-type *CTR3* gene and 825 bp of upstream sequence was amplified by PCR from *H*. *capsulatum* G217B genomic DNA using *CTR3*-specific primers (ORF9-3 and CTR-13) and cloned into a *URA5*-based T-DNA plasmid fusing the *CTR3* gene with sequence encoding a C-terminal FLAG epitope. Either the *CTR3* complementation vector (pDT06) or a control *gfp*-expression vector (pCR628) were transformed by *A*. *tumefaciens*-mediated transformation into the *ctr3* mutants and Ura^+^ transformants were selected by plating on solid HMM.

### Phylogenetic analysis of fungal copper transporters

Reciprocal BLAST searches of fungal genomes using the *H*. *capsulatum* Ctr3 protein sequence and other known fungal copper transporters (e.g., *Saccharomyces* Ctr proteins) were used to identify copper transporter homologs in *Saccharomyces cerevisiae*, *Schizosaccharomyces pombe*, *Candida albicans*, *Neurospora crassa*, *Aspergillus nidulans*, *Aspergillus fumigatus*, *Blastomyces dermatitidis*, *Paracoccidioides braziliensis*, *Magnaporthe oryzae*, *Trichophyton rubrum*, *Ustilago maydis*, and *Cryptococcus neoformans* var *grubii*. Proteins with E-values less than 10^−4^ with and at least 50% coverage were aligned and used for construction of a phylogenetic tree (Clustal Omega).

### Determination of yeast sensitivity to metal ion chelation and toxicity

*H*. *capsulatum* sensitivity to bathocuproine disulfonate (BCS), bathophenanthroline disulfonate (BPS), or excess CuSO_4_ was assayed by addition of two-fold dilutions of chelators or CuSO_4_ to HMM in 48-well plates containing 4 × 10^4^ *H*. *capsulatum* yeasts/ml. Plates were incubated at 37°C with continuous shaking (200 rpm) for 5 days. Yeast proliferation was quantified by measurement of culture turbidity (optical density at 595nm) with a Synergy 2 microplate reader (Biotek). Relative growth in the presence of chelators or CuSO_4_ was determined by normalization of growth to wells lacking BCS, BPS or CuSO_4_. Dose-response curves were determined by non-linear regression of the data and the 50% inhibitory concentrations calculated from the regression curve.

### Intracellular copper or iron measurement by inductively coupled plasma mass spectrometry (ICP-MS)

*Histoplasma* yeasts were pre-grown in HMM to late exponential phase and subsequently treated with 2 mM BCS or 16 μM BPS for 24 h to deplete residual copper or iron carried over from HMM. Thereafter, yeast cells were washed 3 times in PBS to remove BCS or BPS and resuspended in HMM for 3 h or 24 h. Yeast cell concentrations were determined by plating serial dilutions on solid HMM. To measure the intracellular total iron or copper, the yeasts were heated at 95°C to remove all the water. To each yeast sample, 0.1 ml of concentrated ultrapure nitric acid (Fisher Scientific Tracemetal grade distilled at TERL using a Savillex DST-1000) was added. Samples were digested by floating in a 100°C water bath using a floating tube rack for 15–30 min. Samples were visually inspected to ensure complete digestion until no solids were seen. Samples were cooled and diluted by 10-fold in deionized water spiked with 11.11ppb Indium. Final Indium concentration was 10 ppb which was used as an internal standard.

All samples were analyzed on a Thermo Finnigan Element 2 Inductively Coupled Plasma Sector Field Mass Spectrometer. Samples were introduced to the ICP at 100 μl/min using a PFA-100 Microflow self aspirating nebulizer (Elemental Scientific) pumped at 100 μl/min using 0.25 mm I.D. pvc pump tubing and a Gilson 3 peristaltic pump. Iron was measured at m/z 54, 56 and 57 in medium resolution (R = 4000) and copper was measured at m/z 63 and 65 in medium resolution (R = 4000). Calibration standards were prepared by dilution from commercially available single element 1000 μg/ml Fe and 1000 μg/ml Cu (Inorganic Ventures) into 10% v/v nitric acid to match the sample solvent. Calibration standards also included 10 ppb Indium (Inorganic Ventures) as an internal standard. Intracellular total copper or iron were calculated based on 10^8^ yeast cells.

### *CTR* gene expression determination

*CTR* gene transcriptional analyses were determined by quantitative RT-PCR (qRT-PCR) or by *CTR3* promoter-*gfp* reporter fusions. For qRT-PCR, wild-type yeasts or mycelia were grown in HMM containing low (10 nM) or high (10 μM) concentrations of CuSO_4_. RNA was isolated from fungal cells by mechanical disruption with 0.5 mm glass beads, extraction with RiboZol (Amresco) and alcohol precipitation of nucleic acids. Following DNA removal with DNase, RNA was reversed transcribed with Maxima reverse transcriptase (Thermo Scientific) primed with random pentadecamers. Quantitative PCR was carried out using *CTR* gene specific primer pairs with SYBR green-based visualization of product amplification (Bioline). Changes in *CTR* transcript levels relative to actin (*ACT1*) were determined using the *ΔΔ*Ct method [50] after normalization of cycle thresholds to that of the *TEF1* gene.

For construction of the *CTR3* promoter-*gfp* fusion, 1453 bp of sequence upstream of the *CTR3* coding sequence was used to drive *gfp* transcription (P_*CTR3*_-*gfp*; pMK32, [32]). For normalization purposes, a similar *gfp* reporter fusion was created using 661 bp of sequence upstream of the *TEF1* gene (P_*TEF1*_-*gfp)*. Constructs were cloned into a Ura5+ T-DNA vector and transformed by *A*. *tumefaciens*-mediated transformation into the WU15 *ura5* auxotroph. For relative quantification of *CTR3* expression, GFP-fluorescence of P_*CTR3*_-*gfp* transformed yeasts was normalized to GFP-fluorescence of P_*TEF1*_-*gfp* transformed yeasts grown in identical conditions (in vitro and in vivo). For population-based measurements in multi-well plates, GFP fluorescence (485/20 nm excitation, 528/20 nm emission) was measured and the *TEF1* or *CTR3* promoter activity was calculated by normalization to the number of yeasts present (OD595). *CTR3* promoter activity was then normalized to that of the constitutively *TEF1* promoter. For determination of the GFP-fluorescence of individual yeasts, 0.1% Uvitex 3BSA was added to yeast suspensions to label the cell wall and yeasts were examined by microscopy (Nikon E400). GFP-fluorescent (480/30 nm excitation, 535/40 nm emission) and Uvitex-fluorescent images (350/50 nm excitation, 460/50 nm emission) were captured with identical exposure settings and the average GFP fluorescence contained within the Uvitex cell outline was measured (Micro-manager Studio v1.4.5) and ImageJ [51]. Relative *CTR3* promoter activity was determined as the ratio of P_*CTR3*_ GFP fluorescence of individual yeasts to the average P_*TEF1*_ GFP fluorescence.

### Estimation of phagosome copper concentrations

A standard curve of *CTR3* promoter activity at different copper concentrations was generated by incubation of *H*. *capsulatum* yeasts with *TEF1* or *CTR3* promoter-*gfp* fusions in 3M medium with a gradient of copper concentrations. After 48 hours, GFP fluorescence and culture turbidity (OD595) were measured. *TEF1* and *CTR3* promoter activities were determined per OD595 unit, and the *CTR3* promoter activity was normalized to that of the constitutively expressed *TEF1* gene. The actual available copper in the 3M-based media was determined using inductively coupled plasma mass spectrometry (ICP-MS). Media samples were introduced into a Perkin Elmer Nexion 350D ICP-UCT mass spectrometer at the speed of 400 μl/min after being spiked with 10 ppb indium as an internal standard. Copper was measured in DRC (dynamic reaction cell) mode using ammonia gas (0.35 ml/min) to reduce polyatomic and molecular overlaps. Copper concentration analysis showed that 3M medium without any copper addition contained 60 nM copper. Copper concentrations lower than 60 nM were achieved by adding increasing amounts of BCS. For copper concentrations above 60 nm, a curve was fit to the data by four-variable non-linear regression.

For determination of *CTR3* promoter activity in macrophages, macrophages were infected with P_*TEF1*_-*gfp* or P_*CTR3*_-*gfp* yeasts at an MOI of 1:2 in 6-well microtiter plates. Phagocytes were previously seeded into 6-well plates at 5 × 10^5^ (P388D1, RAW 264.7, J774.1, and THP-1) or 3 × 10^6^ (peritoneal macrophages, and BMDMs). Due to the limited yield of alveolar macrophages, 3 × 10^4^ alveolar macrophages were seeded into 96-well plates. For activated macrophages, BMDMs were treated with IFN-γ (200 U/ml and 1000 U/ml), TNF-α (20 U/ml and 100 U/ml) or GM-CSF (2 ng/ml or 10 ng/ml) for 24 hours before infection. 48 hours following macrophage infection, extracellular yeasts were removed by washing macrophages with PBS and intracellular yeasts released by lysis of macrophages with 1% Triton-X100. Yeasts in the macrophage lysates were stained by addition of 0.1% Uvitex and the GFP fluorescence of individual yeasts determined by microscopy and fluorescence quantification. Phagosomal copper concentrations were estimated by comparison of the *CTR3* promoter activity of yeasts recovered from macrophages to the in vitro fluorescence versus copper concentration standard curve.

For determination of the phagosome copper concentration in vivo, C57BL/6 mice were intranasally infected with 2 × 10^4^ P_*TEF1*_-*gfp* or P_*CTR3*_-*gfp* yeasts. At day 6, 10 and 14 post-infection, intracellular yeasts were recovered by euthanizing mice, collection and homogenization of lungs in water, and filtration of the homogenate through a 70 μM cell strainer to remove large debris. The filtrate was treated with collagenase and DNAse for 60 minutes. 0.1% Uvitex was added to the filtrate to label yeasts and the GFP-fluorescence of individual yeast was imaged and measured by microscopy. For IFN-γ treatment of mice, infected mice received 2000 U of IFN-γ intranasally at days 4 and 5 post-infection, with control mice receiving PBS in parallel. At day 6 post-infection, intracellular yeasts were recovered from lung homogenates and the GFP-fluorescence measured by microscopy as above.

### Intramacrophage proliferation of *H*. *capsulatum* yeasts

Macrophage monolayers were established in 96-well plates by seeding with 3 × 10^4^ (P388D1), 2 × 10^5^ (peritoneal macrophages) or 1 × 10^5^ (BMDMs) cells. For experiments with activated macrophages, BMDMs were treated with IFN-γ (1000 U/ml), TNF-α (100 U/ml) or both for 24 hours before infection. Macrophages were then infected with Ctr3-expressing or Ctr3-deficient *H*. *capsulatum* yeasts at an MOI of 1:2. Yeast-infected macrophages were incubated at 37°C for up to 72 hours. At different time points, intracellular yeasts were quantified by removal of any extracellular yeasts with the culture supernatant followed by lysis of the macrophages with sterile H_2_O and plating of the macrophage lysate on solid HMM to enumerate *H*. *capsulatum* CFU.

### Murine model of pulmonary histoplasmosis

To measure in vivo fitness of Ctr3-deficient yeasts compared to Ctr3-expressing yeasts, wild type C57BL/6 mice were infected with *H*. *capsulatum* by intranasal delivery of approximately 2 × 10^4^ yeast cells consisting of equal numbers of Ctr3-expressing (GFP-negative) and Ctr3-deficient yeasts (GFP-fluorescent). Actual numbers and ratios of yeasts delivered were determined by plating serial dilutions of the inocula on solid media for enumeration of CFU. For fungal burden determination at 6, 9, 12, 15, 18 and 21 days post-infection, mice were euthanized, lungs were collected and homogenized in HMM, and serial dilutions of the homogenates were plated on solid HMM to determine the fungal burden (CFU) and fluorescence of recovered colonies. Colony fluorescence was determined with a modified transilluminator and image capture system [52]. The competitive index was calculated as the number of fluorescent colonies (*ctr3*) divided by the number of non-fluorescent colonies (*CTR3*). For testing complementation of the *ctr3* mutant in vivo, the competition assay was repeated using the *ctr3/CTR3* complemented strain and wild type at day 6, 10 and 14 post-infection.

### Quantification and statistical analyses

Data were tabulated and analyzed by Student’s *t*-test (Prism v5, GraphPad Software) for determination of statistically significant differences which are indicated in graphs with asterisk symbols (*, *P* < 0.05; **, *P* < 0.01; *** *P* < 0.001). Dose-response curves were generated by four-variable non-linear regression. The number of mice and the number of biological replicates used in experiments is specified in the relevant figure legends.

### Ethical statements

*H*. *capsulatum* G217B (ATCC 26032) and *H*. *capsulatum* G186A (ATCC 26029) were obtained from American Type Culture Collection (ATCC). All animals were housed in Ohio State University’s AAALAC- and OLAW-accredited animal research facilities (OLAW assurance # A3261-01). All experiments involving mice followed standards in the Public Health Service (PHS) “Guide for the Care and Use of Laboratory Animals” and were approved by The Institutional Animal Care and Use Committee (IACUC) at Ohio State University (protocol # 2007A0241). Mice were anesthetized by inhalation of isoflurane and were euthanized by CO2 inhalation as described in the AVMA "Guidelines for the Euthanasia of Animals”

## Supporting information

S1 Fig The *H*. *capsulatum* genome encodes homologs of Ctr1, Ctr2, and Ctr3 copper transporter families. Putative and proven copper transporter proteins of *Saccharomyces cerevisiae* (Sce), *Schizosaccharomyces pombe* (Spo), *Candida albican*s (Cal), *Neurospora crassa* (Ncr), *Aspergillus nidulans* (Ani), *Aspergillus fumigatus* (Afu), *H*. *capsulatum* (Hca), *Blastomyces dermatitidis* (Bde), *Paracoccidioides braziliensis* (Pbr), *Magnaporthe oryzae* (Mor), *Trichophyton rubrum* (Tru), *Ustilago maydis* (Uma), and *Cryptococcus neoformans* (*grubii*) (Cne) were aligned with Clustal Omega and a Neighbor-joining phylogenetic tree constructed to infer relatedness. Clades were identified representing the Ctr1 (blue), Ctr2 (green), and Ctr3 (red) homologs. Predictions of hypothetical (Hyp), or metal transporters (transporter) from genomic sequencing efforts are listed with gene designation numbers. Protein accession numbers are listed in brackets.
(PDF)

S2 Fig The Ctr3 requirement for yeast proliferation in macrophages and under copper limited conditions extends to a second phylogenetic species of *H*. *capsulatum* (G186A). Growth of the Ctr3-expressing parent strain (*CTR3*, black) and a strain in which the ctr3 gene was deleted (*ctr3Δ*, red) of the G186A genetic background in macrophages **(A)** or in liquid media with limited copper **(B)**. **(A)** A strain in which the *CTR3* locus was deleted was generated by allelic replacement [53] with a hygromycin expression cassette flanked by 2 kb upstream and downstream of the *CTR3* gene. Intracellular *CTR3* and *ctr3* mutant *H*. *capsulatum* yeasts were quantified over 72 hours following infection of P388D1 macrophages (MOI 1:1). Macrophages were lysed, the intracellular yeasts recovered, and the lysate plated on solid HMM medium to enumerate colony forming units (CFU). Data represent the average intramacrophage CFU ± standard deviation among infections with biological replicates (n = 3). Statistically significant differences between *CTR3* and *ctr3* proliferation at each day were determined by one-tailed Student’s *t*-test and are indicated with asterisks (* *P* < 0.05). **(B)** Growth of Ctr3-expressing (*CTR3*, black) and the *ctr3* mutant (*ctr3*, red) strains grown in liquid HMM with the copper chelator BCS were determined by measurement of yeast metabolic conversion of resazurin to fluorescent resorufin (quantified by fluorescence: 530 nm excitation and 590 nm emission 90 minutes following addition of 1 mM resazurin) after 5 days of growth at 37°C. Relative growth was determined by normalization of yeast-dependent resazurin metabolism for each BCS concentration to that of yeasts grown in the absence of BCS. Dose-response curves were generated by non-linear regression and the IC_50_ for BCS treatment of *CTR3* and *ctr3* strains determined as 2,156 μM and 263 μM, respectively. Data represent average growth ± standard deviation among biological replicates (n = 3).
(PDF)

S3 Fig BCS cation chelation is specific for copper. Growth of Ctr3-deficient yeasts (*ctr3*) in BCS-containing media with and without supplementation with copper **(A)**, zinc **(B)** or iron **(C).** Ctr3-deficient yeasts were incubated at 37°C in liquid HMM (black symbols) or HMM containing 100 μM BCS (red symbols) with (squares) and without (circles) 100 μM CuSO_4_ (+Cu), 100 μM ZnSO_4_ (+Zn) or 100 μM FeSO_4_ (+Fe). Yeast growth was monitored by culture turbidity (optical density at 595 nm) over 6 days. Data represent the average growth ± standard deviation among biological replicates (n = 3).
(PDF)

S4 Fig Zinc and iron do not regulate the *CTR3* promoter. *CTR3* promoter activity in 1 μM CuSO_4_ (low *CTR3* promoter activity, black bars) or 10 nM CuSO_4_ (high *CTR3* promoter activity, red bars) with high and low concentrations of zinc **(A),** iron **(B),** or the iron-specific chelator BPS **(C)**. **(A)** *H*. *capsulatum* yeasts were incubated in 3M medium (containing 4 μM FeSO_4_ and 1 μM CuSO_4_ or 10 nM CuSO_4_) with different ZnSO_4_ concentrations (0.3 μM to 64 μM). **(B)** *H*. *capsulatum* yeasts were incubated in 3M medium (containing 4 μM ZnSO_4_ and 1 μM CuSO_4_ or 10 nM CuSO_4_) with different FeSO_4_ concentrations (0.3 μM to 64 μM). **(C)** *H*. *capsulatum* yeasts were incubated in 3M medium (containing 4 μM ZnSO_4_ and 1 μM CuSO_4_ or 10 nM CuSO_4_) with different BPS concentrations (0 μM to 16 μM). The *CTR3* promoter activity was assessed by fluorescence of wild-type yeasts with the *CTR3* promoter-*gfp* fusion (P_*CTR3*_) after normalization to yeasts with the *TEF1* promoter-*gfp* fusion (P_*TEF1*_) grown in identical conditions. After 72 hours incubation at 37°C, culture turbidity (optical density at 595nm) and GFP fluorescence (485 nm excitation, 528 emission) were measured. *TEF1* or *CTR3* promoter activity (GFP fluorescence) was normalized to the yeast density (OD_595_) and the *CTR3* promoter activity then compared to that of the constitutively expressed *TEF1* promoter. Data represent the average relative *CTR3* promoter activity ± standard deviation among biological replicates (n = 3).
(PDF)

S5 Fig Reactive oxygen and pH stresses do not regulate the *CTR3* promoter. *CTR3* promoter activity in 1 μM CuSO_4_ (low *CTR3* promoter activity, black bars) or 10 nM CuSO_4_ (high *CTR3* promoter activity, red bars) at a range of H_2_O_2_ concentrations **(A)** and pH **(B)**. **(A)** *H*. *capsulatum* yeasts were incubated in 3M medium (containing 1 μM CuSO_4_ or 10 nM CuSO_4_) with H_2_O_2_ (0 μM to 250 μM). **(B)** *H*. *capsulatum* yeasts were incubated in 3M medium (containing 1 μM CuSO_4_ or 10 nM CuSO_4_) buffered to different pH with MES (4.5 to 6.0) or HEPES (6.5 to 7.0). The *CTR3* promoter activity was assessed by fluorescence of wild-type yeasts with the *CTR3* promoter-*gfp* fusion (P_*CTR3*_) after normalization to yeasts with the *TEF1* promoter-*gfp* fusion (P_*TEF1*_) grown in identical conditions. After 72 hours incubation at 37°C, culture turbidity (optical density at 595nm) and GFP fluorescence (485 nm excitation, 528 emission) were measured. *TEF1* or *CTR3* promoter activity (GFP fluorescence) was normalized to the yeast density (OD_595_) and the *CTR3* promoter activity then compared to that of the constitutively expressed *TEF1* promoter. Data represent the average relative *CTR3* promoter activity ± standard deviation among biological replicates (n = 3).
(PDF)

S6 Fig *CTR3* complementation of the *ctr3* mutant rescues the *ctr3* fitness in vivo. Wild-type C57BL/6 mice were infected intranasally with 2×10^4^ yeasts consisting of an equal amount of wild-type *CTR3* (RFP-negative) and *ctr3/CTR3* complemented (RFP-expressing) yeasts. At days 6 and 14 post-infection, the pulmonary fungal burden was measured by collecting lungs and plating lung homogenates on solid media for enumeration of RFP-fluorescent and non-fluorescent colony forming units (CFU). Data points represent the individual ratio of RFP-negative (*ctr3/CTR3*) yeasts and RFP-fluorescent (*CTR3*) yeasts at each time point (n = 3 mice) with horizontal bars representing the average ratio. No significant differences in the ratio of complemented and wild-type yeasts compared to the ratio of the number of yeasts in the inoculum were found by one-tailed Student’s *t*-tests.
(PDF)

S7 Fig IFN-γ activates the *CTR3* promoter in intracellular yeasts but not the *TEF1* and *H2B* promoters used for normalization. **(A)** *TEF1* promoter activity in liquid culture or in BMDMs with and without IFN-γ activation. (**B and C**) *CTR3* promoter activity of intracellular *H*. *capsulatum* yeasts in BMDMs with and without IFN-γ activation. **(A)** *H*. *capsulatum TEF1* promoter activity was measured by fluorescence of the P_*TEF1*_*-gfp* fusion in yeasts cultured in high (10 μM) or low (10 nM) copper media or in BMDMs with and without activation by IFN-γ (1000U/mL). **(B)** *CTR3* promoter activity of intracellular yeasts was measured by the fluorescence produced by the P_*CTR3*_*-gfp* reporter after normalization to *H2B* promoter activity (P_*H2B*_*-gfp*) of a parallel population of intracellular yeasts **(C)** *CTR3* promoter activity of intracellular yeasts was measured by the GFP fluorescence produced by the P_*CTR3*_*-gfp* reporter fusion after normalization to the RFP fluorescence produced by the *P*_*TEF1*_*-rfp* reporter fusion within the same yeast cells. In all experiments, BMDMs were infected with *H*. *capsulatum* yeasts (MOI 1:2) and the fluorescence of intracellular yeasts measured after 48 hours by lysis of macrophages, recovery of yeasts, and measurement of GFP or RFP fluorescence in individual yeasts by microscopy (n > 100 yeasts for each sample). Box plots represent quartiles and median fluorescence of the population with lines showing the 10–90% range of the data. Asterisks indicate significant differences in promoter activity compared to non-activated macrophages (*** *P* < 0.001) using Student’s *t*-test and “ns” indicates no significant difference among the experimental groups (*P* > 0.05) using one-way ANOVA with Tukey's Honest Significant Difference test.
(PDF)

S8 Fig Validation of *CTR*3 promoter activity normalization by housekeeping *H2B* and *TEF1* promoter activities in yeasts within macrophage cells. *CTR3* promoter activity in P388D1 or peritoneal macrophages following normalization to *H2B* (**A**) or *TEF1* (**B**) promoter activity in separate or the same yeast cells, respectively. Cell line (P388D1) and primary (peritoneal macrophages (PM)) macrophages were infected with *H*. *capsulatum* yeast (MOI 1:2) and the *CTR3* promoter activity of intracellular yeasts measured by fluorescence of the *P*_*CTR3*_*-gfp* reporter fusion. (**A**) Intracellular yeast GFP fluorescence produced by the *P*_*CTR3*_*-gfp* reporter fusion was normalized to the GFP fluorescence of a population of *P*_*H2B*_*-gfp* reporter fusion yeasts from parallel infections. (**B**) GFP fluorescence produced by the *P*_*CTR3*_*-gfp* reporter fusion was normalized to the RFP fluorescence produced by the *P*_*TEF1*_*-rfp* reporter fusion within the same yeast cell. Data points represent the *CTR3* promoter activity of individual yeasts (n > 100 for each sample) measured by microscopy of intracellular yeasts recovered after lysis of macrophages. Horizontal bars indicate the population mean. Asterisks (*** *P* < 0.001) indicate significant differences in the *CTR3* promoter activity between yeasts recovered from P388D1 cells and from peritoneal macrophages as determined by two-tailed Student’s *t*-test.
(PDF)

S1 Table Histoplasma strains. (PDF)
