# Supplementary material for: Macrophage activation by IFN-γ triggers restriction of phagosomal copper from intracellular pathogens
Source: PLoS Pathog. 2018 Nov 19;14(11):e1007444. doi: 10.1371/journal.ppat.1007444 (PMC6277122; doi:10.1371/journal.ppat.1007444)
Supplement: S1 Fig — Putative and proven copper transporter proteins of Saccharomyces cerevisiae (Sce), Schizosaccharomyces pombe (Spo), Candida albicans (Cal), Neurospora crassa (Ncr), Aspergillus nidulans (Ani), Aspergillus fumigatus (Afu), H. capsulatum (Hca), Blastomyces dermatitidis (Bde), Paracoccidioides braziliensis (Pbr), Magnaporthe oryzae (Mor), Trichophyton rubrum (Tru), Ustilago maydis (Uma), and Cryptococcus neoformans (grubii) (Cne) were aligned with Clustal Omega and a Neighbor-joining phylogenetic tree constructed to infer relatedness. Clades were identified representing the Ctr1 (blue), Ctr2 (green), and Ctr3 (red) homologs. Predictions of hypothetical (Hyp), or metal transporters (transporter) from genomic sequencing efforts are listed with gene designation numbers. Protein accession numbers are listed in brackets. (PDF) [file ppat.1007444.s001.pdf]

Supplemental Figure 1

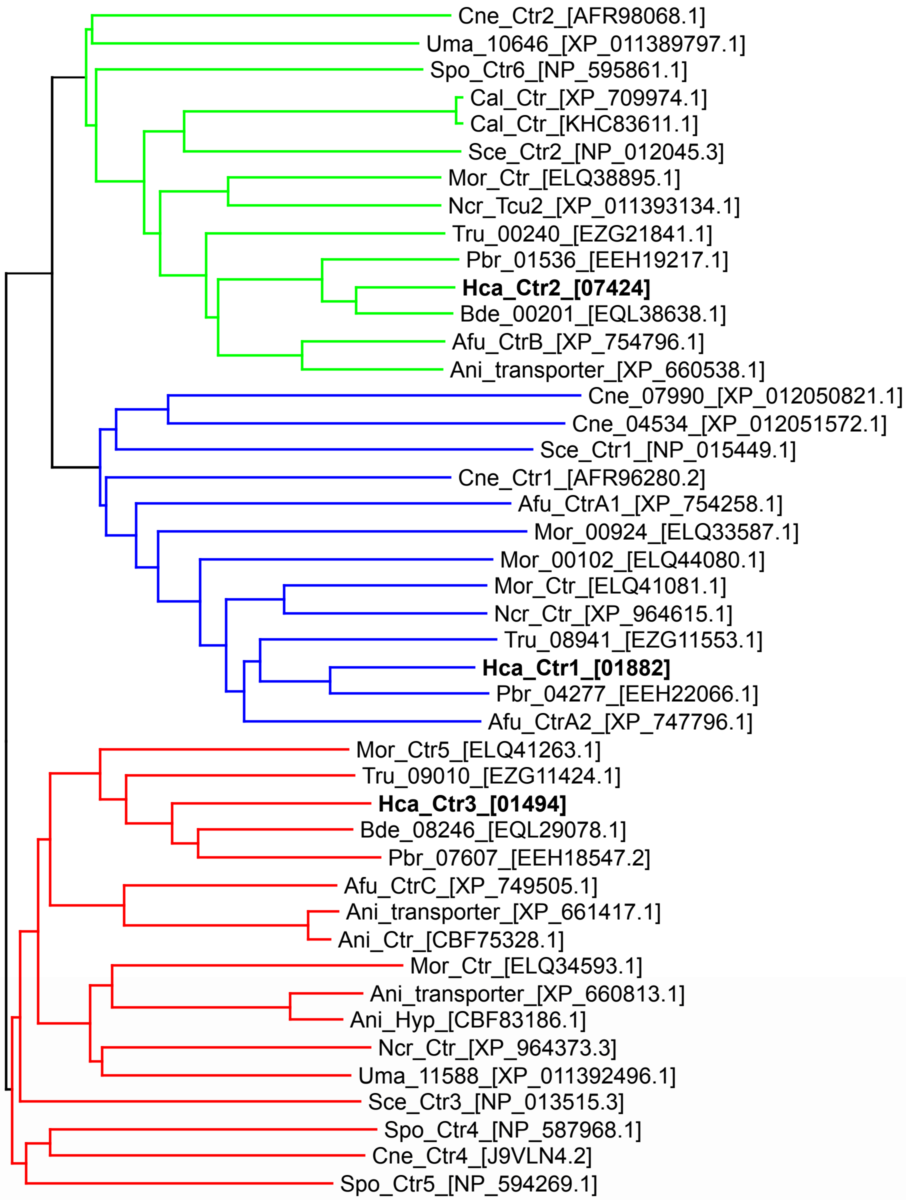

S1 Fig. The *Histoplasma* genome encodes homologs of Ctr1, Ctr2, and Ctr3 copper transporter familes. Putative and proven copper transporter proteins of *Saccharomyces cerevisiae* (Sce), *Schizosaccharomyces pombe* (Spo), *Candida albicans* (Cal), *Neurospora crassa* (Ncr), *Aspergillus nidulans* (Ani), *Aspergillus fumigatus* (Afu), *Histoplasma capsulatum* (Hca), *Blastomyces dermatitidis* (Bde), *Paracoccidioides braziliensis* (Pbr), *Magnaporthe oryzae* (Mor), *Trichophyton rubrum* (Tru), *Ustilago maydis* (Uma), and *Cryptococcus neoformans* var *grubii* (Cne) were aligned with Clustal Omega and a Neighbour-joining phylogenetic tree constructed to infer relatedness. Clades were identified representing the Ctr1 (blue), Ctr2 (green), and Ctr3 (red) homologs. Predictions of hypothetical (Hyp), or metal transporters (transporter) from genomic sequencing efforts are listed with gene designation numbers. Protein accession numbers are listed in brackets.
