# Supplementary material for: Macrophage activation by IFN-γ triggers restriction of phagosomal copper from intracellular pathogens
Source: PLoS Pathog. 2018 Nov 19;14(11):e1007444. doi: 10.1371/journal.ppat.1007444 (PMC6277122; doi:10.1371/journal.ppat.1007444)
Supplement: S2 Fig — Growth of the Ctr3-expressing parent strain (CTR3, black) and a strain in which the ctr3 gene was deleted (ctr3Δ, red) of the G186A genetic background in macrophages (A) or in liquid media with limited copper (B). (A) A strain in which the CTR3 locus was deleted was generated by allelic replacement [53] with a hygromycin expression cassette flanked by 2 kb upstream and downstream of the CTR3 gene. Intracellular CTR3 and ctr3 mutant H. capsulatum yeasts were quantified over 72 hours following infection of P388D1 macrophages (MOI 1:1). Macrophages were lysed, the intracellular yeasts recovered, and the lysate plated on solid HMM medium to enumerate colony forming units (CFU). Data represent the average intramacrophage CFU ± standard deviation among infections with biological replicates (n = 3). Statistically significant differences between CTR3 and ctr3 proliferation at each day were determined by one-tailed Student’s t-test and are indicated with asterisks (* P < 0.05). (B) Growth of Ctr3-expressing (CTR3, black) and the ctr3 mutant (ctr3, red) strains grown in liquid HMM with the copper chelator BCS were determined by measurement of yeast metabolic conversion of resazurin to fluorescent resorufin (quantified by fluorescence: 530 nm excitation and 590 nm emission 90 minutes following addition of 1 mM resazurin) after 5 days of growth at 37°C. Relative growth was determined by normalization of yeast-dependent resazurin metabolism for each BCS concentration to that of yeasts grown in the absence of BCS. Dose-response curves were generated by non-linear regression and the IC50 for BCS treatment of CTR3 and ctr3 strains determined as 2,156 μM and 263 μM, respectively. Data represent average growth ± standard deviation among biological replicates (n = 3). (PDF) [file ppat.1007444.s002.pdf]

Supplemental Figure 2

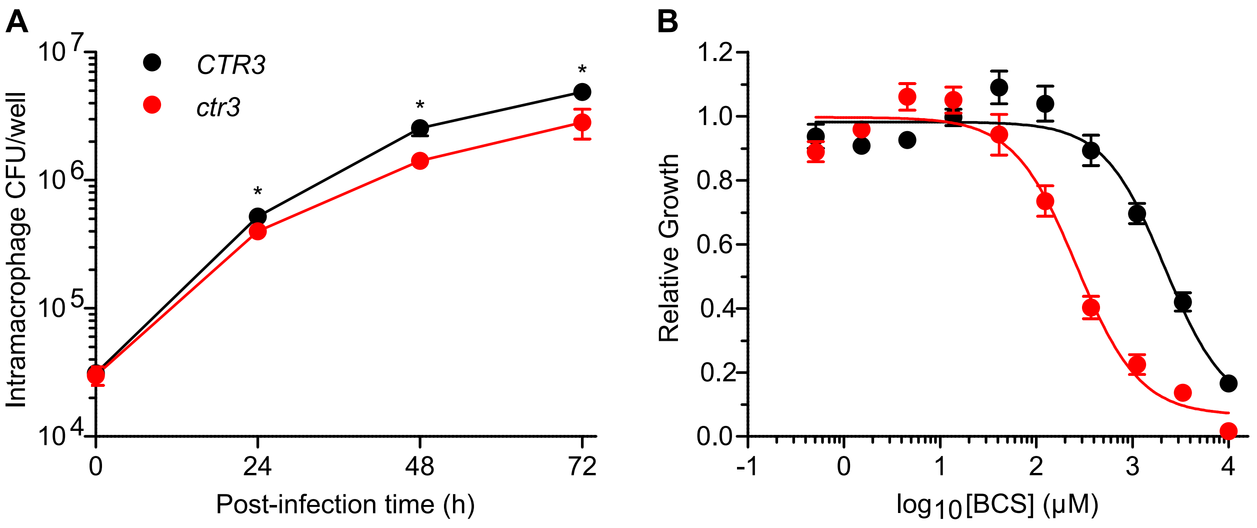

S2 Fig. The Ctr3 requirement for yeast proliferation in macrophages and under copper limited conditions extends to a second phylogenetic species of *H. capsulatum* (G186A). Growth of the Ctr3-expressing parent strain (*CTR3*, black) and a strain in which the *ctr3* gene was deleted (*ctr3Δ*, red) of the G186A genetic background in macrophages **(A)** or in liquid media with limited copper **(B)**. **(A)** A strain in which the CTR3 locus was deleted was generated by allelic replacement (Sebghati et al., 2000) with a hygromycin expression cassette flanked by 2 kb upstream and downstream of the *CTR3* gene. Intracellular *CTR3* and *ctr3* mutant *H. capsulatum* yeasts were quantified over 72 hours following infection of P388D1 macrophages (MOI 1:1). Macrophages were lysed, the intracellular yeasts recovered, and the lysate plated on solid HMM medium to enumerate colony forming units (CFU). Data represent the average intramacrophage CFU ± standard deviation among infections with biological replicates (n = 3). Statistically significant differences between *CTR3* and *ctr3* proliferation at each day were determined by one-tailed Student's *t*-test and are indicated with asterisks (* $P < 0.05$). (B) Growth of Ctr3-expressing (*CTR3*, black) and the *ctr3* mutant (*ctr3*, red) strains grown in liquid HMM with the copper chelator BCS were determined by measurement of yeast metabolic conversion of resazurin to fluorescent resorufin (quantified by fluorescence: 530 nm excitation and 590 nm emission 90 minutes following addition of 1 mM resazurin) after 5 days of growth at 37°C. Relative growth was determined by normalization of yeast-dependent resazurin metabolism for each BCS concentration to that of yeasts grown in the absence of BCS. Dose-response curves were generated by non-linear regression and the IC_{50} for BCS treatment of *CTR3* and *ctr3* strains determined as 2,156 μM and 263 μM, respectively. Data represent average growth ± standard deviation among biological replicates (n = 3).
