# Supplementary material for: Macrophage activation by IFN-γ triggers restriction of phagosomal copper from intracellular pathogens
Source: PLoS Pathog. 2018 Nov 19;14(11):e1007444. doi: 10.1371/journal.ppat.1007444 (PMC6277122; doi:10.1371/journal.ppat.1007444)
Supplement: S3 Fig — Growth of Ctr3-deficient yeasts (ctr3) in BCS-containing media with and without supplementation with copper (A), zinc (B) or iron (C). Ctr3-deficient yeasts were incubated at 37°C in liquid HMM (black symbols) or HMM containing 100 μM BCS (red symbols) with (squares) and without (circles) 100 μM CuSO4 (+Cu), 100 μM ZnSO4 (+Zn) or 100 μM FeSO4 (+Fe). Yeast growth was monitored by culture turbidity (optical density at 595 nm) over 6 days. Data represent the average growth ± standard deviation among biological replicates (n = 3). (PDF) [file ppat.1007444.s003.pdf]

Supplemental Figure 3

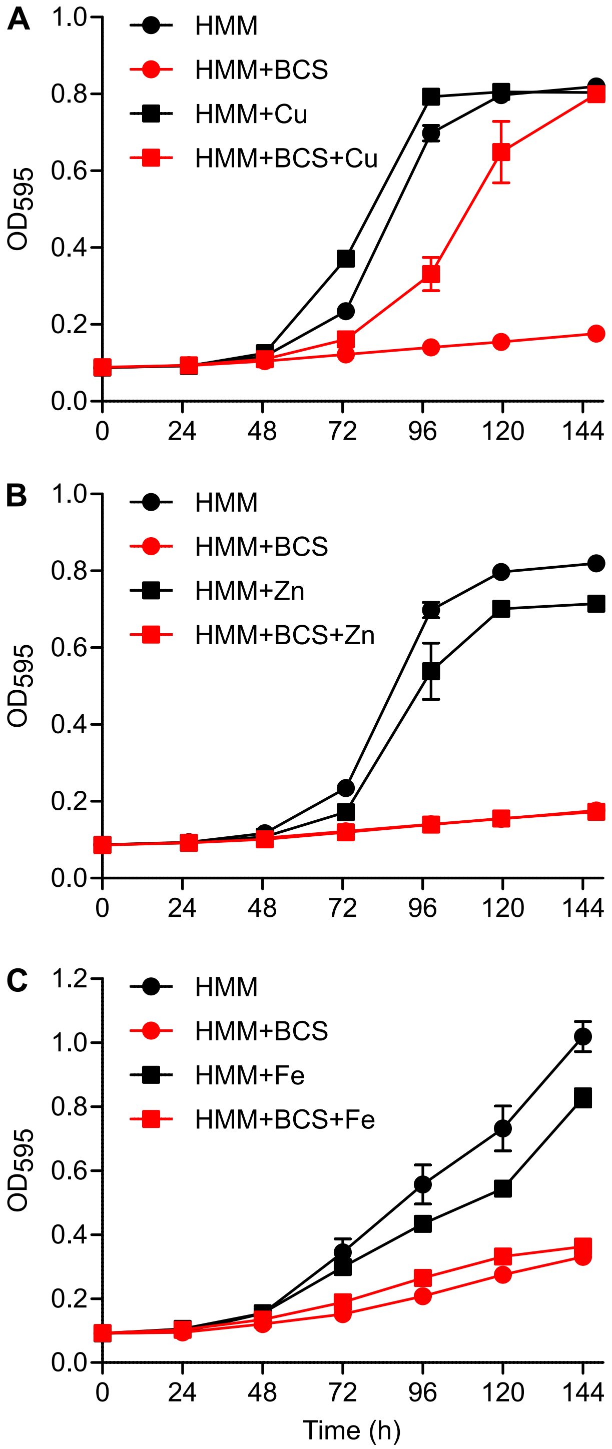

S3 Fig. BCS cation chelation is specific for copper. Growth of Ctr3-deficient yeasts (*ctr3*) in BCS-containing media with and without supplementation with copper **(A)**, zinc **(B)** or iron **(C)**. Ctr3-deficient yeasts were incubated at 37°C in liquid HMM (black symbols) or HMM containing 100 μM BCS (red symbols) with (squares) and without (circles) 100 μM $CuSO_4$ (+Cu), 100 μM $ZnSO_4$ (+Zn) or 100 μM $FeSO_4$ (+Fe). Yeast growth was monitored by culture turbidity (optical density at 595 nm) over 6 days. Data represent the average growth ± standard deviation among biological replicates (n = 3).
