# Supplementary material for: Macrophage activation by IFN-γ triggers restriction of phagosomal copper from intracellular pathogens
Source: PLoS Pathog. 2018 Nov 19;14(11):e1007444. doi: 10.1371/journal.ppat.1007444 (PMC6277122; doi:10.1371/journal.ppat.1007444)
Supplement: S5 Fig — CTR3 promoter activity in 1 μM CuSO4 (low CTR3 promoter activity, black bars) or 10 nM CuSO4 (high CTR3 promoter activity, red bars) at a range of H2O2 concentrations (A) and pH (B). (A) H. capsulatum yeasts were incubated in 3M medium (containing 1 μM CuSO4 or 10 nM CuSO4) with H2O2 (0 μM to 250 μM). (B) H. capsulatum yeasts were incubated in 3M medium (containing 1 μM CuSO4 or 10 nM CuSO4) buffered to different pH with MES (4.5 to 6.0) or HEPES (6.5 to 7.0). The CTR3 promoter activity was assessed by fluorescence of wild-type yeasts with the CTR3 promoter-gfp fusion (PCTR3) after normalization to yeasts with the TEF1 promoter-gfp fusion (PTEF1) grown in identical conditions. After 72 hours incubation at 37°C, culture turbidity (optical density at 595nm) and GFP fluorescence (485 nm excitation, 528 emission) were measured. TEF1 or CTR3 promoter activity (GFP fluorescence) was normalized to the yeast density (OD595) and the CTR3 promoter activity then compared to that of the constitutively expressed TEF1 promoter. Data represent the average relative CTR3 promoter activity ± standard deviation among biological replicates (n = 3). (PDF) [file ppat.1007444.s005.pdf]

Supplemental Figure 5

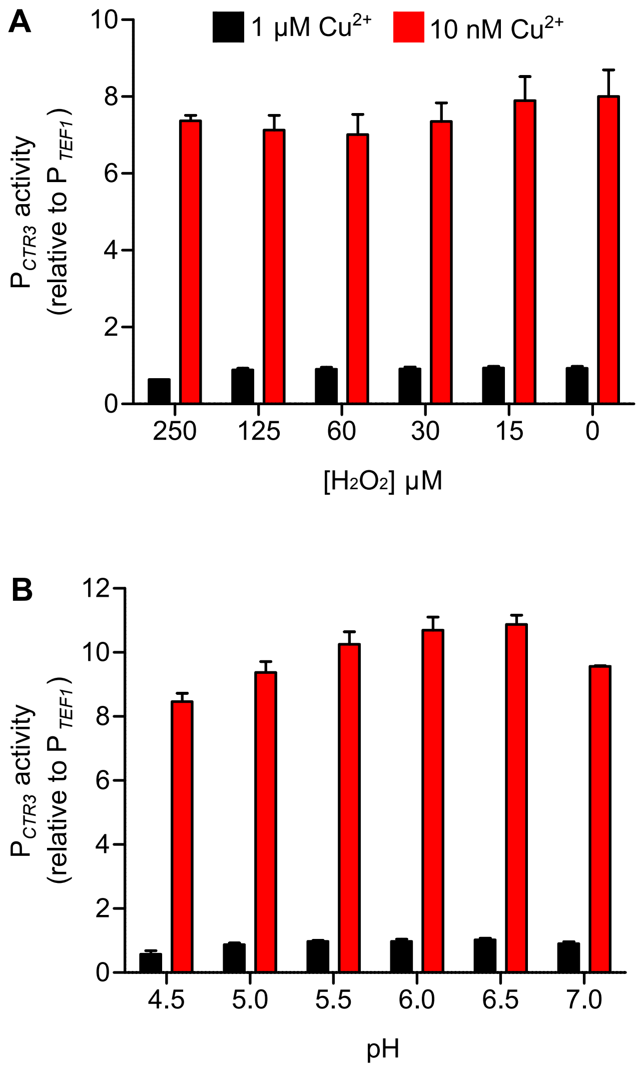

S5 Fig. Reactive oxygen and pH stresses do not regulate the *CTR3* promoter. *CTR3* promoter activity in 1 μM $CuSO_4$ (low *CTR3* promoter activity, black bars) or 10 nM $CuSO_4$ (high *CTR3* promoter activity, red bars) at a range of H_2O_2 concentrations **(A)** or pH **(B)**. **(A)** *H. capsulatum* yeasts were incubated in 3M medium (containing 1 μM $CuSO_4$ or 10 nM $CuSO_4$) with H_2O_2 (0 μM to 250 μM). **(B)** *H. capsulatum* yeasts were incubated in 3M medium (containing 1 μM CuSO4 or 10 nM CuSO4) buffered to different pH with MES (4.5 to 6.0) or HEPES (6.5 to 7.0). The *CTR3* promoter activity was assessed by fluorescence of wild-type yeasts with the *CTR3* promoter-*gfp* fusion (P_{CTR3}) after normalization to yeasts with the *TEF1* promoter-gfp fusion (P_{TEF1}) grown in identical conditions. After 72 hours incubation at 37°C, culture turbidity (optical density at 595nm) and GFP fluorescence (485 nm excitation, 528 emission) were measured. *TEF1* or *CTR3* promoter activity (GFP fluorescence) was normalized to the yeast density (OD_{595}) and the *CTR3* promoter activity then compared to that of the constitutively expressed *TEF1* promoter. Data represent the average relative *CTR3* promoter activity ± standard deviation among biological replicates (n = 3).
