# Supplementary material for: Macrophage activation by IFN-γ triggers restriction of phagosomal copper from intracellular pathogens
Source: PLoS Pathog. 2018 Nov 19;14(11):e1007444. doi: 10.1371/journal.ppat.1007444 (PMC6277122; doi:10.1371/journal.ppat.1007444)
Supplement: S6 Fig — Wild-type C57BL/6 mice were infected intranasally with 2×10^4^ yeasts consisting of an equal amount of wild-type CTR3 (RFP-negative) and ctr3/CTR3 complemented (RFP-expressing) yeasts. At days 6 and 14 post-infection, the pulmonary fungal burden was measured by collecting lungs and plating lung homogenates on solid media for enumeration of RFP-fluorescent and non-fluorescent colony forming units (CFU). Data points represent the individual ratio of RFP-negative (ctr3/CTR3) yeasts and RFP-fluorescent (CTR3) yeasts at each time point (n = 3 mice) with horizontal bars representing the average ratio. No significant differences in the ratio of complemented and wild-type yeasts compared to the ratio of the number of yeasts in the inoculum were found by one-tailed Student’s t-tests. (PDF) [file ppat.1007444.s006.pdf]

Supplemental Figure 6

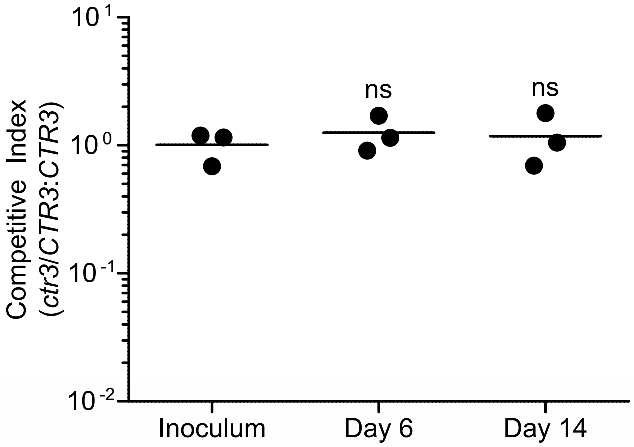

S6 Fig. *CTR3* complementation of the *ctr3* mutant rescues the *ctr3* fitness in vivo. Wild-type C57BL/6 mice were infected intranasally with 2×10^4 yeasts consisting of an equal amount of wild-type *CTR3* (RFP-negative) and *ctr3/CTR3* complemented (RFP-expressing) yeasts. At days 6 and 14 post-infection, the pulmonary fungal burden was measured by collecting lungs and plating lung homogenates on solid media for enumeration of RFP-fluorescent and non-fluorescent colony forming units (CFU). Data points represent the individual ratio of RFP-negative (*CTR3*) yeasts and RFP-fluorescent (*ctr3/CTR3*) yeasts at each time point (n = 3 mice) with horizontal bars representing the average ratio. No significant differences in the ratio of complemented and wild-type yeasts compared to the ratio of the number of yeasts in the inoculum were found by one-tailed Student's *t*-tests.
