# Supplementary material for: Macrophage activation by IFN-γ triggers restriction of phagosomal copper from intracellular pathogens
Source: PLoS Pathog. 2018 Nov 19;14(11):e1007444. doi: 10.1371/journal.ppat.1007444 (PMC6277122; doi:10.1371/journal.ppat.1007444)
Supplement: S7 Fig — (A) TEF1 promoter activity in liquid culture or in BMDMs with and without IFN-γ activation. (B and C) CTR3 promoter activity of intracellular H. capsulatum yeasts in BMDMs with and without IFN-γ activation. (A) H. capsulatum TEF1 promoter activity was measured by fluorescence of the PTEF1-gfp fusion in yeasts cultured in high (10 μM) or low (10 nM) copper media or in BMDMs with and without activation by IFN-γ (1000U/mL). (B) CTR3 promoter activity of intracellular yeasts was measured by the fluorescence produced by the PCTR3-gfp reporter after normalization to H2B promoter activity (PH2B-gfp) of a parallel population of intracellular yeasts (C) CTR3 promoter activity of intracellular yeasts was measured by the GFP fluorescence produced by the PCTR3-gfp reporter fusion after normalization to the RFP fluorescence produced by the PTEF1-rfp reporter fusion within the same yeast cells. In all experiments, BMDMs were infected with H. capsulatum yeasts (MOI 1:2) and the fluorescence of intracellular yeasts measured after 48 hours by lysis of macrophages, recovery of yeasts, and measurement of GFP or RFP fluorescence in individual yeasts by microscopy (n > 100 yeasts for each sample). Box plots represent quartiles and median fluorescence of the population with lines showing the 10–90% range of the data. Asterisks indicate significant differences in promoter activity compared to non-activated macrophages (*** P < 0.001) using Student’s t-test and “ns” indicates no significant difference among the experimental groups (P > 0.05) using one-way ANOVA with Tukey's Honest Significant Difference test. (PDF) [file ppat.1007444.s007.pdf]

Supplementary figure 7

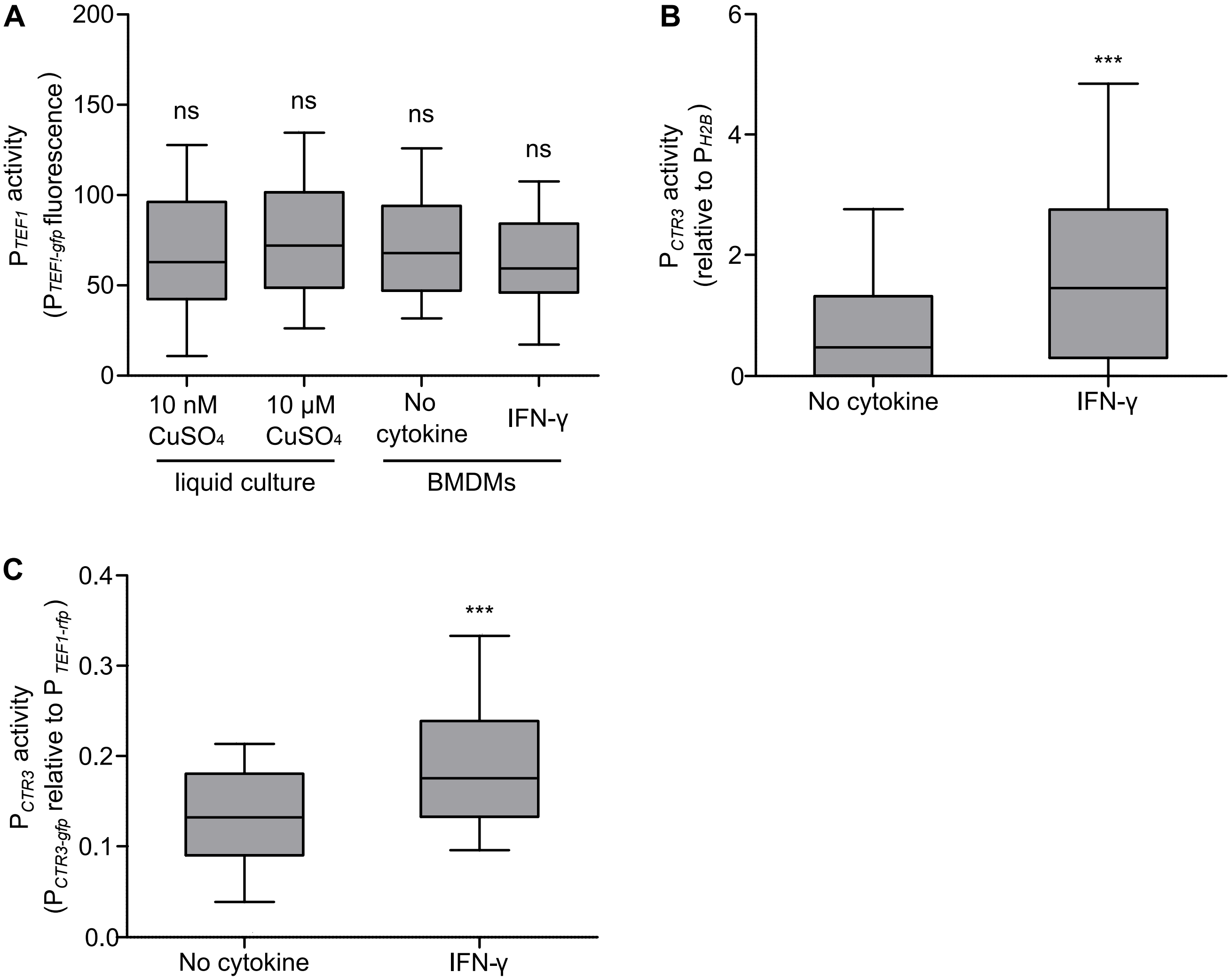

S7 Fig. IFN-γ activates the *CTR3* promoter in intracellular yeasts but not the *TEF1* and *H2B* promoters used for normalization. *TEF1* **(A)** promoter activity in liquid culture or BMDMs and *CTR3* **(B and C)** promoter activity of intracellular *H. capsulatum* yeasts in BMDMs with and without IFN-γ activation. **(A)** *H. capsulatum TEF1* promoter activity was measured by fluorescence of the P_{TEF1}-*gfp* fusion in yeasts cultured in high (10 µM) or low (10 nM) copper media or in BMDMs with and without activation by IFN-γ (1000U/mL). **(B)** *CTR3* promoter activity of intracellular yeasts was measured by the fluorescence produced by the P_{CTR3}-*gfp* reporter after normalization to *H2B* promoter activity (P_{H2B}-*gfp*) of a parallel population of intracellular yeasts. **(C)** *CTR3* promoter activity of intracellular yeasts was measured by the GFP fluorescence produced by the P_{CTR3}-*gfp* reporter fusion after normalization to the RFP fluorescence produced by the P_{TEF1}-*rf*p reporter fusion within the same yeast cells. In all experiments, BMDMs were infected with *H. capsulatum* yeasts (MOI 1:2) and the fluorescence of intracellular yeasts measured after 48 hours by lysis of macrophages, recovery of yeasts, and measurement of GFP or RFP fluorescence in individual yeasts by microscopy (n > 100 yeasts for each sample). Box plots represent quartiles and median fluorescence of the population with lines showing the 10-90% range of the data. Asterisks indicate significant differences in promoter activity compared to non-activated macrophages (*** $P < 0.001$) using Student's *t*-test and "ns" indicates no significant difference among the experimental groups ($P > 0.05$) using one-way ANOVA with Tukey's Honest Significant Difference test.
