# Supplementary material for: Macrophage activation by IFN-γ triggers restriction of phagosomal copper from intracellular pathogens
Source: PLoS Pathog. 2018 Nov 19;14(11):e1007444. doi: 10.1371/journal.ppat.1007444 (PMC6277122; doi:10.1371/journal.ppat.1007444)
Supplement: S8 Fig — CTR3 promoter activity in P388D1 or peritoneal macrophages following normalization to H2B (A) or TEF1 (B) promoter activity in separate or the same yeast cells, respectively. Cell line (P388D1) and primary (peritoneal macrophages (PM)) macrophages were infected with H. capsulatum yeast (MOI 1:2) and the CTR3 promoter activity of intracellular yeasts measured by fluorescence of the PCTR3-gfp reporter fusion. (A) Intracellular yeast GFP fluorescence produced by the PCTR3-gfp reporter fusion was normalized to the GFP fluorescence of a population of PH2B-gfp reporter fusion yeasts from parallel infections. (B) GFP fluorescence produced by the PCTR3-gfp reporter fusion was normalized to the RFP fluorescence produced by the PTEF1-rfp reporter fusion within the same yeast cell. Data points represent the CTR3 promoter activity of individual yeasts (n > 100 for each sample) measured by microscopy of intracellular yeasts recovered after lysis of macrophages. Horizontal bars indicate the population mean. Asterisks (*** P < 0.001) indicate significant differences in the CTR3 promoter activity between yeasts recovered from P388D1 cells and from peritoneal macrophages as determined by two-tailed Student’s t-test. (PDF) [file ppat.1007444.s008.pdf]

Supplementary figure 8

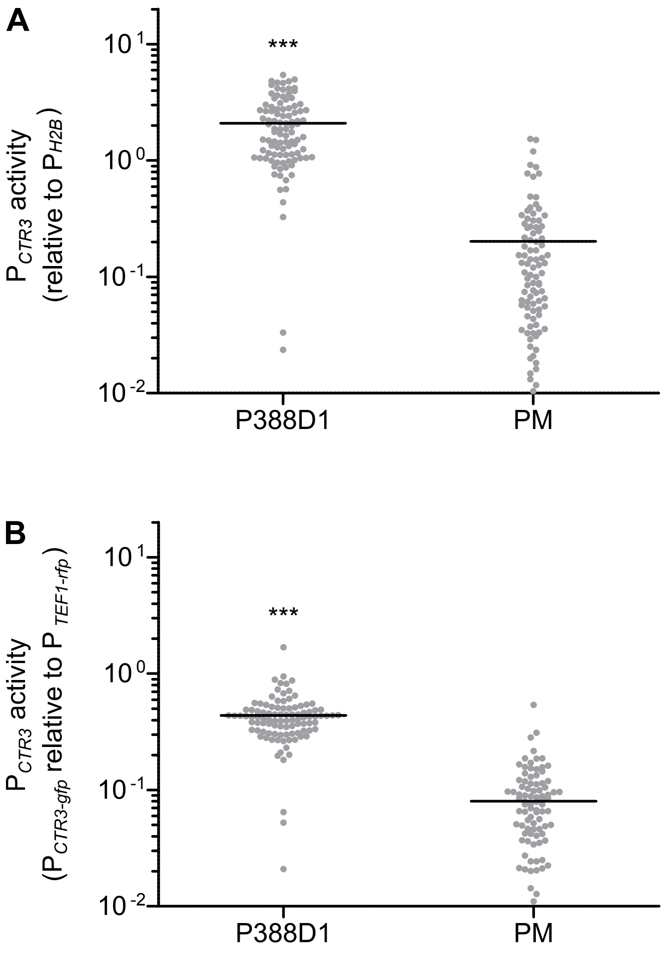


**S8 Fig. Validation of *CTR3* promoter activity normalization by housekeeping *H2B* and *TEF1* promoter activities in yeasts within macrophage cells.** *CTR3* promoter activity in P388D1 or peritoneal macrophages following normalization to *H2B* **(A)** or *TEF1* **(B)** promoter activity in separate or the same yeast cells, respectively. Cell line (P388D1) and primary (peritoneal macrophages (PM)) macrophages were infected with *H. capsulatum* yeast (MOI 1:2) and the *CTR3* promoter activity of intracellular yeasts measured by fluorescence of the $P_{CTR3}$-*gfp* reporter fusion. **(A)** Intracellular yeast GFP fluorescence produced by the $P_{CTR3}$-*gfp* reporter fusion was normalized to the GFP fluorescence of a population of $P_{H2B}$-*gfp* reporter fusion yeasts from parallel infections. **(B)** GFP fluorescence produced by the $P_{CTR3}$-*gfp* reporter fusion was normalized to the RFP fluorescence produced by the $P_{TEF1}$-*rfp* reporter fusion within the same yeast cell. Data points represent the *CTR3* promoter activity of individual yeasts (n > 100 for each sample) measured by microscopy of intracellular yeasts recovered after lysis of macrophages. Horizontal bars indicate the population mean. Asterisks (*** $P < 0.001$) indicate significant differences in the *CTR3* promoter activity between yeasts recovered from P388D1 cells and from peritoneal macrophages as determined by two-tailed Student's *t*-test.
