# Supplementary material for: Macrophage activation by IFN-γ triggers restriction of phagosomal copper from intracellular pathogens
Source: PLoS Pathog. 2018 Nov 19;14(11):e1007444. doi: 10.1371/journal.ppat.1007444 (PMC6277122; doi:10.1371/journal.ppat.1007444)
Supplement: S1 Table — (PDF) [file ppat.1007444.s009.pdf]

Supplemental Table 1: *Histoplasma* Strains

Strain[1,2]	Genotype[3]	Other Designation
WU8[1]	*ura5-32Δ*	*CTR3*
WU15[2]	*ura5-42Δ*	*CTR3*
OSU161[1]	*ura5-32Δ ctr3-1Δ::hph*	*ctr3*
OSU189[1]	*ura5-32Δ zzz::T-DNA(pCR624: URA5, rfp)*	*CTR3*
OSU190[1]	*ura5-32Δ ctr3-1Δ::hph zzz::T-DNA(pCR623: URA5, gfp)*	*ctr3*
OSU216[2]	*ura5-42Δ zzz::T-DNA(pKG06: URA5)*	*CTR3*
OSU233[2]	*ura5-42Δ zzz::pQS01(apt3,* P_{TEF1}*-rfp)*	*CTR3*
OSU264[2]	*ura5-42Δ zzz::pCR628 (URA5,* P_{H2B}*-gfp)*	P_{H2B}*-gfp*
OSU296[2]	*ura5-42Δ zzz::pQS01(apt3,* P_{TEF1}*-rfp) zzz::T-DNA(pKG06: URA5)*	*CTR3*
OSU310[2]	*ura5-42Δ zzz::pQS01(apt3,* P_{TEF1}*-rfp) ctr3-2::T-DNA(pBHt2: hph)*	*ctr3*
OSU311[2]	*ura5-42Δ zzz::pQS01(apt3,* P_{TEF1}*-rfp) ctr3-3::T-DNA(pBHt2: hph)*	*ctr3*
OSU315[2]	*ura5-42Δ zzz::pQS01(apt3,* P_{TEF1}*-rfp) ctr3-2::T-DNA(pBHt2: hph) zzz::T-DNA(pCR628: URA5, gfp)*	*ctr3*
OSU316[2]	*ura5-42Δ zzz::pQS01(apt3,* P_{TEF1}*-rfp) ctr3-2::T-DNA(pBHt2: hph) zzz::T-DNA(pDT06: URA5, CTR3)*	*ctr3/CTR3*
OSU317[2]	*ura5-42Δ zzz::pQS01(apt3,* P_{TEF1}*-rfp) ctr3-3::T-DNA(pBHt2: hph) zzz::T-DNA(pCR628: URA5, gfp)*	*ctr3*
OSU318[2]	*ura5-42Δ zzz::pQS01(apt3,* P_{TEF1}*-rfp) ctr3-3::T-DNA(pBHt2: hph) zzz::T-DNA(pDT06: URA5, CTR3)*	*ctr3/CTR3*
OSU326[2]	*ura5-42Δ zzz::T-DNA(pCR623: URA5,* P_{TEF1}*-gfp)*	P_{TEF1}*-gfp*
OSU327[2]	*ura5-42Δ zzz::T-DNA(pMK32: URA5,* P_{CTR3}*-gfp)*	P_{CTR3}*-gfp*
OSU373[2]	*ura5-42Δ zzz::pQS01 (G418,* P_{TEF1}*-RFP) zzz:pMK32(URA5,* P_{CTR3}*-gfp)*	P_{CTR3}*-gfp /* P_{TEF1}*-rfp*

[1,2] strains were constructed in the *Histoplasma* [1]G186A (ATCC 26029) or [2]G217B (ATCC 26032) backgrounds

[3] gene designations:

zzz::T-DNA: T-DNA integration at an undetermined chromosomal location
hph: hygromycin B phosphotransferase (hygromycin resistance)
apt3: aminoglycoside phosphotransferase (G418 resistance)
gfp: green-fluorescence protein
rfp: red-fluorescence protein (tdTomato)
CTR3: copper transporter
H2B: histone 2B
TEF1: translation elongation factor EF-1α
URA5: orotate phosphoribosyltransferase
